# Pancancer Analysis Revealed the Value of RAC2 in Immunotherapy and Cancer Stem Cell

**DOI:** 10.1155/2023/8485726

**Published:** 2023-05-12

**Authors:** Ranran Liu, Tianyu Li, Guohong Zhang, Yejuan Jia, Jingxuan Liu, Lijia Pan, Yunfeng Li, Chunsheng Jia

**Affiliations:** ^1^School of Acupuncture, Moxibustion and Tuina, Hebei University of Chinese Medicine, Shijiazhuang 050200, China; ^2^Hebei Key Laboratory of Chinese Medicine Research on Cardiocerebrovascular Disease, Hebei University of Chinese Medicine, Shijiazhuang 050200, China

## Abstract

**Objective:**

To investigate the oncogenic effect and clinical significance of RAC2 in pancarcinoma from the perspective of tumor immunity and cancer stem cell.

**Methods:**

After in-depth mining of TCGA, GEO, UCSC, and other databases, basic information of the RAC2 gene and its expression in tumor tissues as well as the relationship between RAC2 and tumor were analyzed based on survival, mutation, immune microenvironment, tumor stemness, and enrichment analysis on related pathways.

**Results:**

RAC2 mRNA expression was increased in most tumor tissues and was associated with their prognosis. Compared to normal tissues, the RAC2 mutation rate was higher in patients with skin melanoma, uterine sarcoma, and endometrial cancer. RAC2 had a strong relation with immune cell infiltration, immunomodulators, immunotherapy markers, cancer stem cell of THYM, and immune-related pathways.

**Conclusions:**

This study explored the potential importance of RAC2 in the prognosis, immunotherapy, and cancer stem cell of 33 cancers, laying the foundation for mechanistic experiments and its future application in clinical practice. However, the results using bioinformatics methods could be affected by the differences in patients across databases. Thus, the present results were preliminary and required further experimental validation.

## 1. Introduction

Cancer is a major cause of death all over the world. The most common cancers include lung, colorectal, liver, gastric, and breast cancers [1]. The rise of new incidence and mortality from cancer and its impact on social life have attracted widespread attention. Tumor bioinformatics database has promoted the development of basic cancer research and improved clinical efficacy. Due to the diversity and complexity of tumorigenesis mechanisms, the specific role of many genes in tumors is not clear. Therefore, it is of particular significance to comprehensively analyze the expression and mechanism of these genes and the possibility of clinical application. RAC2 (Ras-associated C3 botulinum toxin substrate 2) is a small-signal GTP enzyme. Small G proteins are a class of low molecular weight proteins, which are molecular switches in cells mainly distribute in the cytoplasm or in the inner plasma membrane, and can catalyze the interconversion between GTP and GDP [2]. It is composed of more than 150 members. These members can be divided into five subfamilies including Ras, Rho, Rab, Arf/Sar, and Ran [3]. RAC proteins are small G proteins of the Rho family that participates in many regulatory pathways, including in gene expression, cell proliferation, cell adhesion, and cytoskeleton formation [4], which mainly include three different subfamilies, namely, RAC1, RAC2, and RAC3 [5]. RAC1 has been extensively studied in the Rac family, and it has been shown to regulate cellular communication in tumors. RAC1 affects tumor cell proliferation, migration, and metabolism by regulating important target molecules in PAK kinase [6, 7], cascade signaling pathway [8], and glycolytic signaling pathway [9]. Rac2 is expressed exclusively in blood-derived cells [10]. It has been shown to have key regulatory roles in regulating cell signaling and actin-based cytoskeleton formation [11], mainly localized in the phagosomal membrane and can be involved in hematopoietic cell formation [12]. There are few studies on the role of RAC2 on tumors, and the molecular mechanisms are not clear. Some experimental studies on RAC2 mainly focus on individual tumor types, and the existing studies do not comprehensively examine multiple tumor types at the same time to determine their similarities and differences. Using expression data from 33 human cancers in large databases such as TCGA, RAC2 expression, survival, mutation, immune microenvironment, and enrichment analysis in various tumors can be explored. This study preliminarily revealed the relationship of RAC2 in tumor immunity and explored its potential mechanism so as to provide a basis for further study of the relationship between RAC2 and tumor.

## 2. Materials and Methods

### 2.1. Overview of Basic Gene Information

We first performed the mapping of genes with RAC2 expression levels in various tissues and cells under a normal state. The genomic map information for the RAC2 gene in the UCSC genome browser December 2013 (GRCh38/HG38) Assembly [13] was obtained; meanwhile, we collected the gene dialogue used to visualize RAC2 in different animals in the UCSC Genome Browser. After logging in the online HPA (Human Protein Atlas) database and entered “RAC2” to query the status of RAC2 expression in different cells and tissues under normal conditions, low specificity was considered as “ NX (standardized expression) “ in at least one tissue/region/cell type ≥ 1 but not elevated in any of them.

### 2.2. mRNA and Protein Expression Analysis

We observed the difference of RAC2 expression between adjacent normal tumor tissues and specific tumor subtypes of different tumors or TCGA projects (the short version of each tumor is given in Figure S1) in TIMER2. To reduce the errors caused by the algorithm from the database, “Expression Analysis-Box Plot” module of the GEPIA2 network server was used to obtain a box plot of expression difference between tumor tissue and corresponding normal tissue in the GTEx (genotype-tissue expression) database under the criteria of the *P* value =0.01 and |log2FC (fold change)| > 1 with “matching TCGA normal tissue and GTEx data”. After clicking the “CPTAC” module of UALCAN (The University of Alabama at Birmingham Cancer data analysis Portal), we entered “RAC2” and selected the available data sets of 10 tumors for analysis. In addition, a protein expression map of RAC2 was obtained based on proteomics expression profiles at various pathological stages at UALCAN (^∗^*P* < 0.05, ^∗∗^*P* < 0.01, and ^∗∗∗^*P* < 0.001).

### 2.3. Survival Prognosis Analysis

We explored the survival differences between the high- and low-expressed RAC2 groups using the Kaplan-Meier method to evaluate the RAC2 value in prognosis. We investigated the relation between RAC2 expression and overall survival (OS), disease-specific survival (DSS), and progression-free survival (PFI) using R language and survival package on a uniform standardized pancancer dataset downloaded from the TCGA database with multivariate Cox regression analyses. After calculating hazard ratio (HR), 95% confidence intervals, and log-rank *P* values, we generated Kaplan-Meier survival plots. If the hazard ratio was greater than 1 (HR > 1), this suggested that RAC2 expression was the promoter of death events.

### 2.4. Gene Variation Analysis

The genetic variation, mutation frequency, mutation type, and copy number change (CNA) of RAC2 in the TCGA database were detected on the cBioPortal website. In the “Cancer Type Summary” module, the mutation frequency, mutation type, and copy number change (CNA) of all cancers in the TCGA database were analyzed. With the “mutation” module construction, we obtained information about the RAC2 mutation sites indicated in the schematic diagram of the protein structure. In the “Plots” module, we collected differences in overall survival, sex, and age of onset between the different subtypes of RAC2-non-mutant cancers and RAC2-mutant cancers. Summary data on the OS, DSS, disease-free survival (DFS), and progression-free survival (PFS) of RAC2 patients with and without mutations in the TCGA tumor database were obtained from the “comparison” module. Finally, a survival map was generated.

### 2.5. Potential Association Analysis with Immune-Related Factors

We downloaded data on the relation between the RAC2 mRNA expression and the expression of immunomodulators [14] (chemokines, receptors, MHCs, immunosuppressants, and immunostimulants) and immune checkpoint [15] (inhibitors and stimuli) from the TCGT database to explore their potential association. Four results with the highest relevance were shown in a scatter plot. Studies have demonstrated that [16] biomarkers such as TMB, MSI, and neoantigens are closely related to the immune reaction. Therefore, we also study the relationship of RAC2 expression with these indicators in various tumors. Next, the matrix score and immune score (http://bioinformatics.mdanderson.org/) were calculated for each case using the ESTIMATE package. The ESTIMATE algorithm [17] includes matrix score, immune score, and estimation score.

### 2.6. Relationship between Cancer Stem Cell and Rac2 mRNA Expression

We analyzed cancer stem cells by tumor stemness. The RNA tumor stemness score was calculated by mRNA features on the Sangerbox website and then integrated with RAC2 expression data for each sample. Expression profile data (GSE158997) of THYM was obtained from the GEO Dataset module on NCBI, the corresponding platform data was GPL16686, and then, ID was transformed with R software. Differential analysis was then performed using the R software package limma (version 3.40.6) to obtain differentially expressed genes (DEGs) between the different comparison groups and the control groups. The volcano map of the DEGs was drawn with the R software. The top 50 DEG was visualized by R software. The coexpression networks in DEGs were constructed by WGCNA [18] based on the scale-free topology criteria. We then used the R software to analyze all DEG and determine the soft threshold power to construct a weighted coexpression network, clustering DEG into several modules with different color labels. The modules most relevant to THYM were determined as the key modules to further enrich the analysis and get key genes. The stemness signatures and consensus clustering of the key module genes were developed and achieved from StemChecker (http://stemchecker.sysbiolab.eu/) [19]. We then analyzed the molecular correlation to obtain the relation between RAC2 and key cancer stemness genes in THYM.

### 2.7. Rac2-Related Gene Enrichment Analysis

The previous study found a robust correlation between RAC2 and ACC, BRCA, COAD, KICH, TGCT, and UVM, and GSEA (gene set enrichment analysis) was used to study the functional and pathway differences of RAC2 between low- and high-expressed RAC2 groups in these cancers. Predefined gene sets were obtained from the autologous MSigDB database. The top five related signaling pathways of NES (normalized enrichment score) were presented in the form of a graph. We also performed GO (Gene Ontology) enrichment analysis, first obtained the functional annotation chart data of BP (biological process), CC (cellular component), and MF (molecular function), conducted the enrichment analysis with clusterProfiler package (version 3.14.3), and then used ggplot2 to produce a chart for the top group in *P* value (except for MF in BRCA).

### 2.8. Immunohistochemistry Verification

Using the HPA database (Human Protein Atlas), we obtained IHC (immunohistochemistry) images to show the RAC2 protein expression in cancers and the corresponding normal tissues. To further validate the reliability of previous data, we purchased three tissue microchips of breast cancer and their matching paratumoral tissues from Shaanxi Ernan Biological Co., Ltd. After dewaxing and hydration, samples were first placed in citrate buffer for pressure cooker antigen thermal repair and then incubated at 4°C with RAC2 antibody (DF-6273, affinity) overnight. After washing by PBS, the slices were incubated with HRP-labeled goat anti-rabbit IgG polymer secondary antibody (PV-6001; ZSGB-BIO) for 20 minutes (min) at 37°C and washed by PBS three times (3 min a time), and fresh DAB color development solution (ZLI-9018) was added for 3 min before observation under a microscope. Next, 1% hydrochloride alcohol differentiation solution was added and then removed after 4 s, the samples were rinsed under tap water for 3 min, added with the return blue liquid, and removed after 4-20 s. Again, the samples were then rinsed under tap water for 3 min. Gradient alcohol dehydration was then performed. Finally, the neutral tree gum sheet was sealed, and the results were observed under a microscope.

## 3. Results

### 3.1. Overview of Basic Gene Information

**Figure 1:**
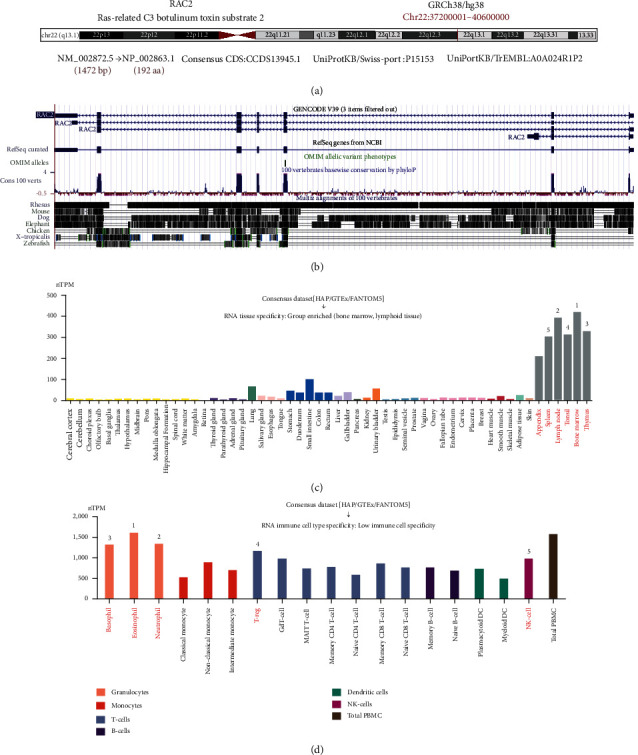
Structural characteristics of RAC2 in different species and the levels of RAC2 expression in different tissues and cells under normal physiological states. (a) Genomic localization of the human RAC2 gene. (b) Visualization of RAC2 gene conservation analysis in different animals. (c) Expression analysis of the RAC2 genes in different tissues. (d) Consensus datasets in different blood cells using HPA, Monaco, and Schmiedel expression analysis.

To investigate the basic information of human RAC2 (NM_002872.5 for mRNA or NP_002863.1 for protein; Figure 1(a)). RAC2 is located at positions 37200001-40600000 on chromosome 22. As shown in Figure 1(b), the RAC2 protein structure is conserved in different vertebrates (such as human, chicken, and monkey). We then determined the expression of RAC2 in tissues, organs, and cells under physiological state. As shown in Figure 1(c), RAC2 was expressed in all detected tissues in the dataset in HPA, GTEx, and FANTOM5 (consistent standardized expression value > 1). RAC2 gene expression was the highest in bone marrow, followed by lymph node, thymus, tonsil, and spleen. We also analyzed the expression of RAC2 in different immune cells, and a low immune cell specificity was observed (Figure 1(d)). The results suggested that RAC2 was the highest in eosinophils, followed by neutrophils, basophils, T-regs, and NK cells.

### 3.2. mRNA and Protein Expression Analysis

**Figure 2:**
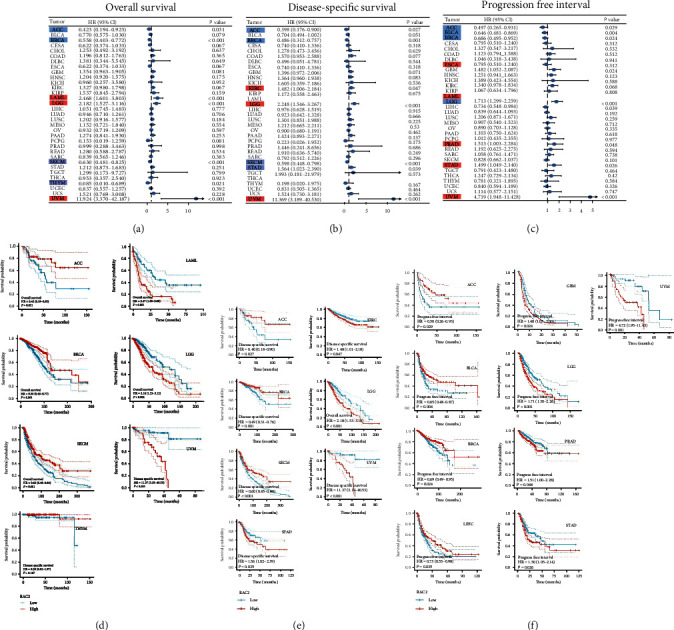
Survival analysis of RAC2 in pancancer. Prognostic analysis of RAC2 by multivariate Cox regression: (a) the observation of the overall survival of 33 tumors by RAC2; (b) the observation of disease-specific survival in 33 tumors by RAC2; (c) results of RAC2 on pancancer progression interval; (d) cancers with a significant prognostic association with RAC2 in pancancer overall survival; (e) cancer where RAC2 is significantly associated with prognosis in pancancer disease-specific survival; (f) cancers with a significant prognostic association with RAC2 in the pancancer progression-free interval period.

The expression of RAC2 in 33 tumors of TCGA was determined. Differentially expressed RAC2 between cancer and adjacent normal tissues was obtained from “Gene_DE” module of TIMER2. As shown in Figure 2(a), in 9 cancer tissues, the expression of RAC2 was higher than that of corresponding normal tissues. In LUAD, LUSC (all *P* < 0.001), KICH, PRAD, STAD, and THCA (all *P* < 0.01), the RAC2 expression was lower than that of corresponding normal tissues. We further evaluated RAC2 expression in different cancers by supplementing normal tissues from the GTEx dataset, and 16 kinds of cancer were significantly increased in tumors with statistical differences (Figure 2(b)). Here, the CPTAC database was used to determine RAC2 expression at the protein level close to the direct manifestation of the disease, and it contained a large number of clinical data to verify the relationship between protein and clinical manifestation. The results of the CPTAC database filtered the cancers of BRCA, COAD, KIRC, GBM, HNSC, LIHC, PAAD, and UCEC (all *P* < 0.01). The total protein expression of RAC2 in tissues was higher than that in normal tissues (Figure 2(c)). RNA levels (transcription levels) and protein levels (translation levels) were consistent, and the results from the two databases corroborated each other. We also observed the relationship between RAC2 expression and cancer pathological stage. RAC2 expression in ACC, KICH, KIRC, PAAD, SKCM, STAD, and THCA all showed a *P* < 0.05 and was associated with the pathological stage of the cancers (Figure 2(d)).

### 3.3. Survival Analysis Data

**Figure 3:**
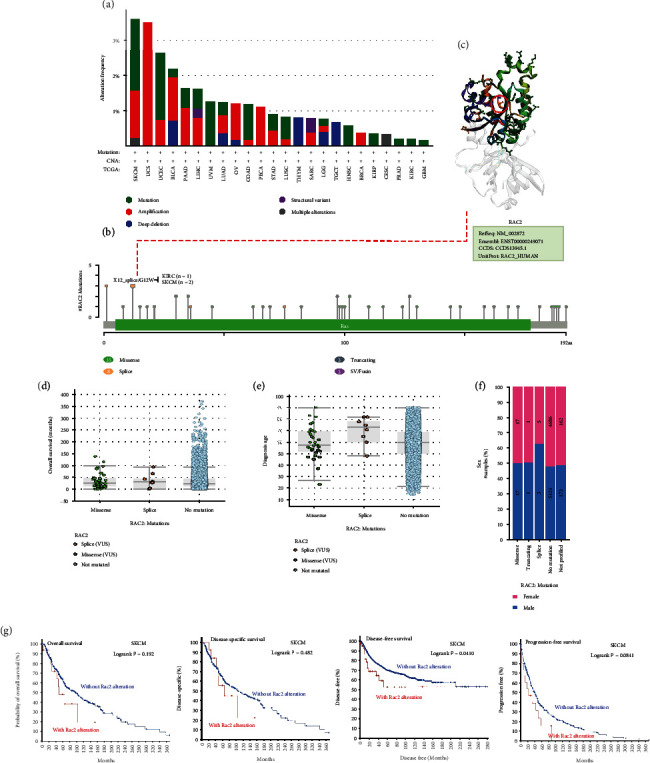
Mutational signature of the RAC2 gene in different tumor tissues of TCGA: (a) mutation frequency and mutation type; (b) mutant site of RAC2; (c) in the 3D structure of RAC2, the frequency of the mutation site alteration is the highest (X12_splice/G12W); (d) relationship between RAC2 mutation and overall survival; (e) relationship between RAC2 mutation and age of onset; (f) relationship between RAC2 mutation and sex; (g) correlation between the RAC2 mutation and the OS, DSF, DFS, and PFS rates of SKCM.

The relationship between RAC2 gene expression and the prognosis of cancer patients was analyzed from OS, DSS, and PFI. OS results showed a significant correlation (Figure 3(a)) between RAC2 and ACC, BRCA, LAML, LGG, SKCM, THYM, and UVM. DSS results showed significant prognostic relation between RAC2 and ACC, BRCA, KIRC, LGG, SKCM, STAD, and UVM (Figure 3(b)). PFI results demonstrated a significant prognosis correlation between RAC2 and ACC, BLCA, BRCA, GBM, LGG, LIHC, PRAD, STAD, and UVM (Figure 3(c)). These outcomes indicated that low RAC2 expression may be associated with poor prognosis in 5 cancers; however, high RAC2 expression may be associated with poor prognosis in 6 cancers. Finally, we screened the tumor types with RAC2 gene expression and OS for further study.

### 3.4. Analysis of Gene Variation

Human cancers develop as a result of the accumulation of genetic alterations. Therefore, we analyzed RAC2 gene variation in pancancer from the TCGA cohort. As shown in Figure S2a, RAC2 showed the highest change frequency (*P* > 3%) in cutaneous melanomas with “mutation” and “amplification” as the dominant types. In the cases of UCS, amplification was the major form, with an alteration frequency of approximately 3.5%. RAC2 changes in THYM and TGCT were mainly characterized by deep deletion, whereas all RAC2 changes in CESC were multiple alterations. In conclusion, RAC2 changes were characterized by “mutation” and “amplification” in most cancers (Figure 3(a)). The types, sites, and cases of RAC2 mutations are shown in Figure S2b. The results indicated that “missense mutations” were the predominant type of RAC2 gene alterations and that X12_splice/G12W alterations (detected in KIRC and SKCM) induced configuration (Figure 3(b)). In the TCGA cohort, the OS of RAC2 missense-mutant cancers (median, 26.83; interquartile range, 12-48) and RAC2 splicing-mutant cancers (median, 30.61; interquartile range, 9.75-59.81) were not statistically different from those with RAC2 nonmutant disease (median, 23.59; interquartile range, 12.59-44.91). The diagnosis age of RAC2 missense-mutant cancer patients (median,5 7.5; interquartile range, 51.75-69) and RAC2 splicing-mutant cancer patients (median,73; interquartile range, 61.25-81) was not statistically different from the those with RAC2 nonmutant disease (median, 60; interquartile range, 25-69). In terms of sex, patients with RAC2 missense-mutant cancer (female, 50%; male, 50%), RAC2 splicing-mutant cancer (female, 37.5%; male, 62.5%), and RAC2 nonmutant cancer (female,52.23%; male,47.77%) were statistically different; however, because of the small number of cases, the reliability of the results cannot be fully confirmed. Furthermore, we examined the association between RAC2 genetic mutations and clinical survival outcomes in various cancers. As shown in Figure 3(d), the DFS prognosis was better in SKCM patients without RAC2 modification. However, compared with the RAC2 changes, the OS, DSS, and DFS were not significantly different.

### 3.5. Analysis of Data of Immune Infiltration

**Figure 4:**
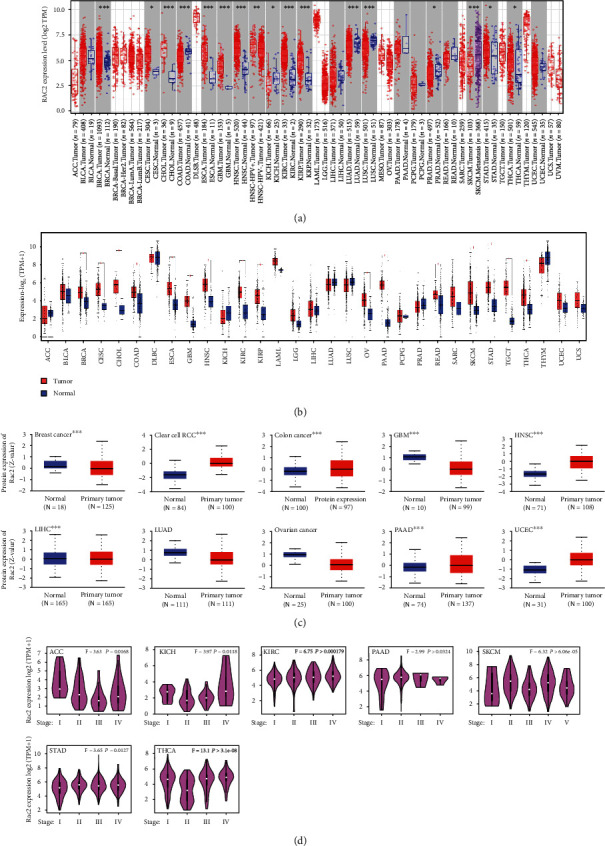
RAC2 mRNA expression and protein levels in human cancers. (a) The RAC2 mRNA expression in different cancer or some cancer subtypes and the corresponding adjacent tissues was analyzed by TIMER2. (b) Box plots comparing RAC2 expression levels in 10 cancers (TCGA project) relative to control tissues (GTEx database). (c) The expression levels of total RAC2 protein in normal tissues versus BRCA, KIRC, COAD, GBM, and HNSC. (d) Stage-dependent expression levels of RAC2. According to TCGA data, the mRNA expression levels of RAC2 at different pathological stages of 7 cancers.

**Figure 5:**
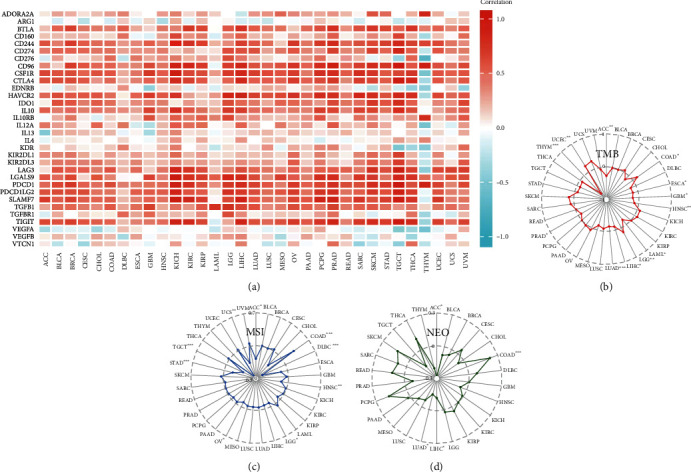
The relationship between (Figure 7) RAC2 expression and the immune checkpoint, TMB, MSI, and NEO in pancancer.

**Figure 6:**
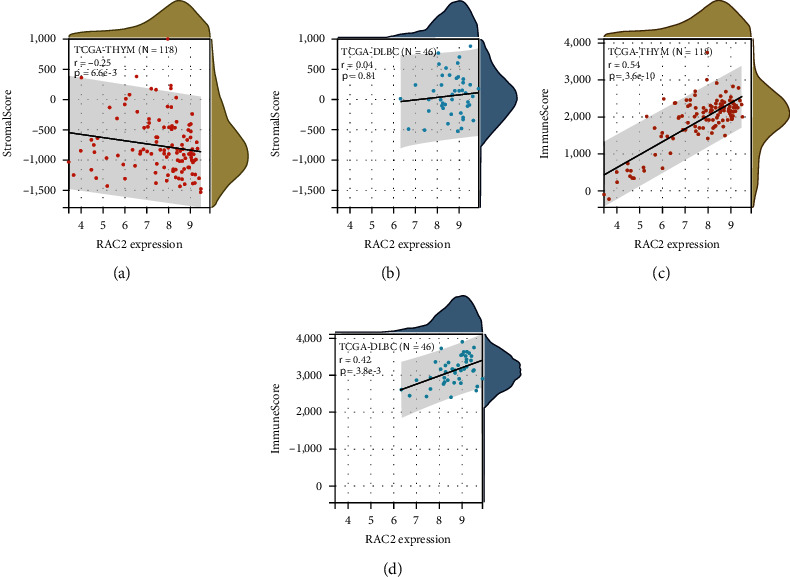
Correlation between the RAC2 expression and the ESTIMATE score.

**Figure 7:**
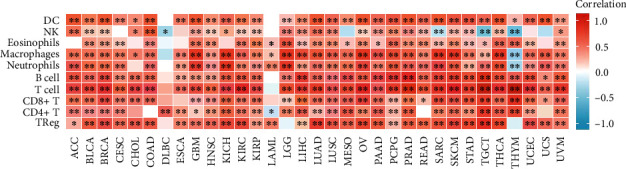
ssGSEA analysis of the relationship between RAC2 levels and immune infiltration in the TME.

**Figure 8:**
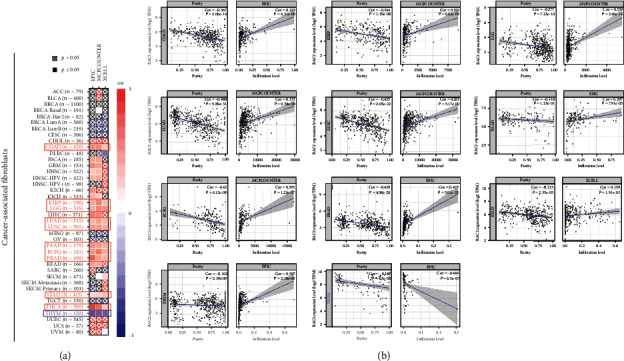
Different algorithms were used to explore the potential correlation between the RAC2 gene expression levels and the levels of cancer-associated fibroblast infiltration in different types of cancer tissues.

**Figure 9:**
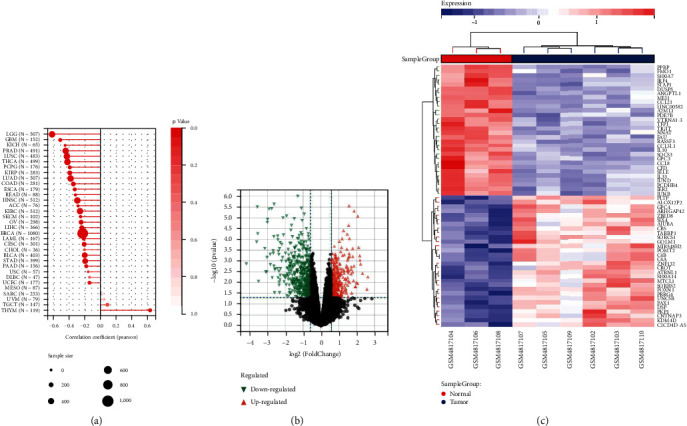
We compared the correlation between RAC2 and the stemness index of each tumor and the expression profile of DEGs between THYM and normal subjects by RNA tumor stemness score. (a) RAC2 was positively associated with tumor stemness in THYM and related to LGG, GBM, and KICH. (b) Volcano plot of differential gene expression levels between THYM versus controls. (c) Top 50 differential genes between THYM and controls.

High expression of RAC2 in immune tissues, organs, and cells could be found in Figure 4; thus, it was speculated that the role of RAC2 in antitumor may be correlated to immunity. Positive associations of RAC2 with large amounts of immunomodulators were found (Figure S3). In chemokines and their receptors, the expression of RAC2 was positively correlated with CCL4 in KICH, CCL4, CCL5, and CXCL9 in TGCT.  Figure 5 shows the relationship between RCA2 and MHC genes, and RAC2 expression was positively associated with HLA-DRA, HLA-DQA1, HLA-DPA1, and HLA-DPB2 in KICH. In the immune checkpoint inhibitors (Figure 6(a)), RAC2 expression was positively associated with LGALS9 in KICH, CD96, TIGIT, and PDCD1 in TGCT. Furthermore, RAC2 expression was positively related to TMB in COAD but negatively connected with TMB in 9 cancers (Figure 6(b)). RAC2 was positively associated with MSI in COAD and negatively associated with MSI in ACC, DLBC, HNSC, LGG, OV, STAD, TGCT, and UCS (Figure 6(c)). We then investigated the relation between RAC2 and NEO (Figure 6(d)). The data showed that the upregulated RAC2 levels were significantly correlated with the number of neoantigens in COAD. The correlation between RAC2 levels and immune scores was calculated, and we estimated the stromal scores and immune scores for estimate scores in 33 cancer types. Among 33 cancers, 32 of them were statistically significant, among which DLBC was not statistically significant; moreover, the stromal score of TGCT was negatively correlated with RAC2 expression (Figure 7). The top three tumors with a significant association between RAC2 and stromal scores were LGG, KICH, and GBM. The top three tumors with a significant association between RAC2 and immune scores were TGCT, LGG, and KICH (Figure S4). Furthermore, to elucidate the relation between RAC2 and the immune cells in the pancancer of TME, the ssGSEA algorithm was applied to determine the immune cell abundance on the corresponding datasets. The results showed that RAC2 maintained a close association with these immune cells in 33 cancers. In particular, elevated RAC2 maintained a significant positive relationship with CD4 + T cells, CD8 + T cells, NK cells, neutrophils, and macrophages (Figure 8). The details of these immune cells in the various algorithms are shown in additional Figure S3-8. We also analyzed the relationship between fibroblasts and RAC2 and found that RAC2 was positively associated with COAD, LUAD, and PCPG and was negatively associated with THYM (Figure 9).

### 3.6. Relationship between RAC2 Expression and Cancer Stem Cell

**Figure 10:**
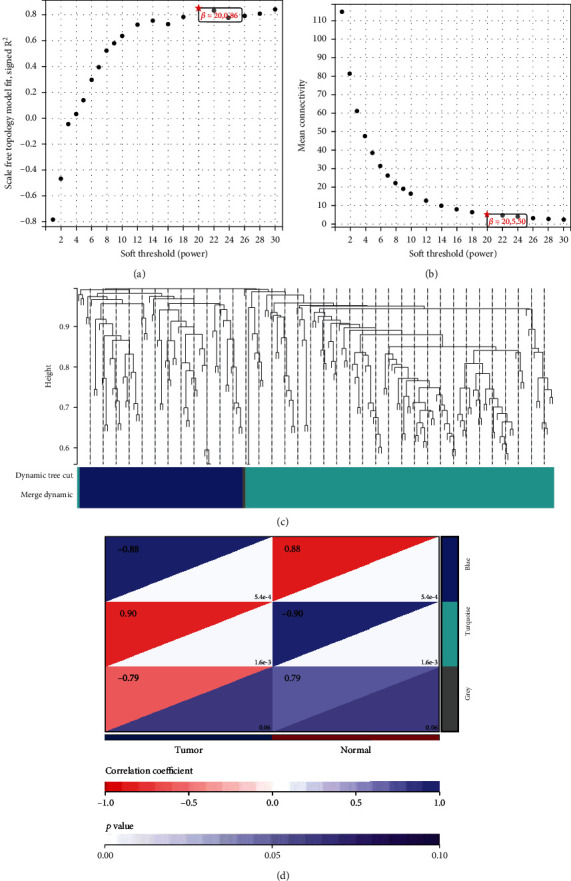
WGCNA of differential genes in THYM versus controls. (a) Estimation of the soft thresholds for the scale-free coexpression networks. (b) Average connectivity. (c) Cluster dendrogram of all differential genes. (d) Correlation between each module and THYM patients or controls.

**Figure 11:**
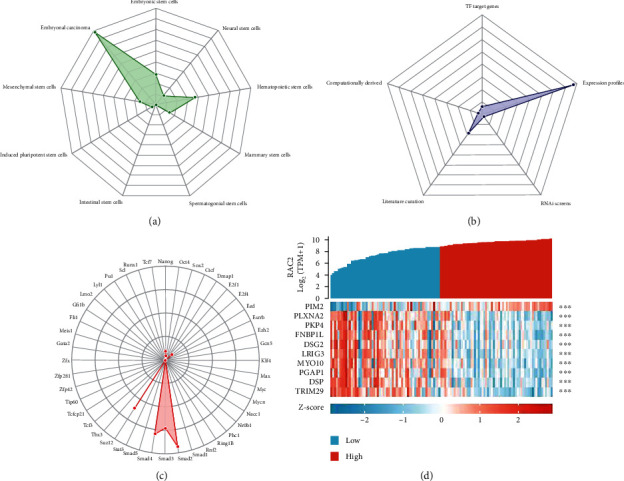
The overlap between genes in key gene modules and stem cell types is shown in the overlapping radar chart. (a) The statistical detail table displays the significance of the enrichment of genes included in composite gene sets for the different stem cell types among the key genes found in StemChecker. (b) The significance of gene enrichment of selected stem traits in key module genes found in StemChecker. (c) The significance of gene enrichment as transcription factor targets among key module genes identified in StemChecker. (d) Molecular correlation of the top ten RAC2 with the key tumor stemness genes for THYM. *P* value was calculated by the hypergeometric test to assess the degree of enrichment of the fully annotated human genome. The *P* value was adjusted with Bonferroni correction to show genetic overlap between key gene modules and stem cell types.

Cancer stem cell (CSC) refers to cells in tumors with the ability to self-renew and produce heterogeneous tumor cells [20]. The stem cell index (stemness indices) is an indicator to describe the similarity between tumor cells and stem cells. mRNAsi is an indicator that describes the degree of similarity of tumor cells to stem cells and can be considered as a quantification of CSCs [21]. From Figure 9(a), it can be seen that RAC2 was significantly and positively related with the tumor stemness of THYM and was significantly negatively associated with LGG, GBM, and KICH. As shown in Figure 9, RAC2 was significantly positively associated with tumor stemness in THYM and negatively related with LGG, GBM, and KICH. We therefore further investigated the THYM and found 853 differential genes in THYM compared with normal people; specifically, 524 were downregulated, and 329 were upregulated (Figure 9(b)). The top 50 differential genes were shown in Figure 9(c). After further processing of the identified 853 DEGs with the WGCNA package in the R software, we built a scale-free coexpression network (scale-free *R*2 > 0.86) with a soft threshold power of 20. In the subsequent analysis, the soft threshold weight *β* was set to 20 as the scale independence reached 0.86 (Figure 10(a)) and had a relatively close average connectivity (Figure 10(b)). DEGs were clustered into 3 modules, and the smallest module was 30. The DEGs were shown in Figure 10(c). We calculated and plotted the correlation between each module and the THYM (Figure 10(d)). The results showed that blue (-0.88) and turquoise (0.9) were the most negative and positive modules associated with THYM, respectively. Among them, the turquoise module, including 129 DEGs, was considered the key modules associated with THYM. The top 50 DEGs are in the turquoise module. We processed these key module genes with the StemChecker tool. The composite gene set of different cell types was the combination of all selected stemness traits of the corresponding cell type. A total of 26 THYM key tumor stem genes in THYM were obtained by including gene enrichment (Figure 11(a)) in different stem cell types, key module genes, and stem gene enrichment (Figure 11(b)). The key module genes were the transcription factor targets (Figure 11(c)). We then analyzed the molecular correlation of RAC2 with these 26 genes; the top ten correlations were shown in Figure 11(d).

### 3.7. Enrichment Analysis of RAC2-Related Partners

**Figure 12:**
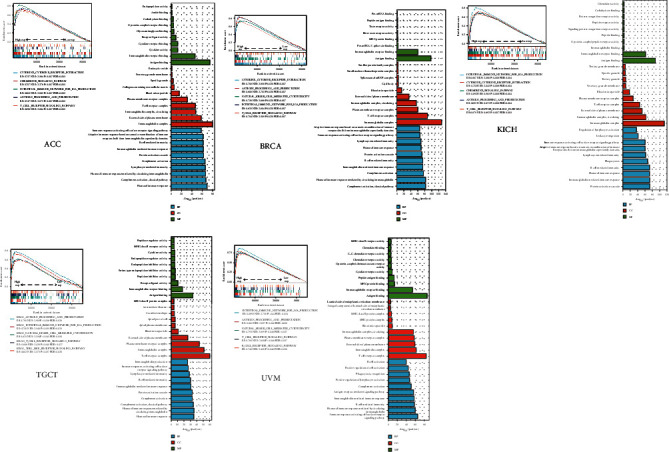
The KEGG/GO enrichment pathway analysis for ACC, BRCA, COAD, KICH, TGCT, and UVM.

To further study the molecular mechanisms of RAC2 in tumorigenesis, we performed a series of pathway enrichment analyses on ACC, BRCA, COAD, KICH, TGCT, and UVM. As shown in Figure 12, KEGG pathway analysis showed that RAC2 may be involved in “cytokine receptor interaction,” “chemokine signaling pathway,” “antigen processing and presentation,” and other related pathways in the process of tumor pathogenesis. In addition, GO enrichment data further indicated that differential genes were enriched for immunity in a single tumor, such as “humoral immune response mediated by calculating immunoglobulin” in BP, “immunoglobulin complex” in CC, and “antigen binding” in MF. It is interesting that the “intestinal immune network for iga production” pathway was simultaneously enriched in the GO enrichment analysis and KEGG pathway analysis, suggesting that RAC2 may also affect gut function.

### 3.8. Histochemical Results

**Figure 13:**
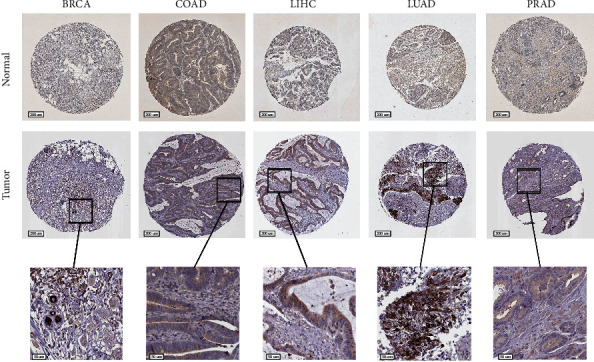
Results of immunohistochemical staining of the RAC2 gene in the HPA database in five tumors and their corresponding adjacent tissues. The expression of the RAC2 gene was significantly higher in BRCA, COAD, LIHC, LUAD, and PRAD than in the corresponding normal tissues.

**Figure 14:**
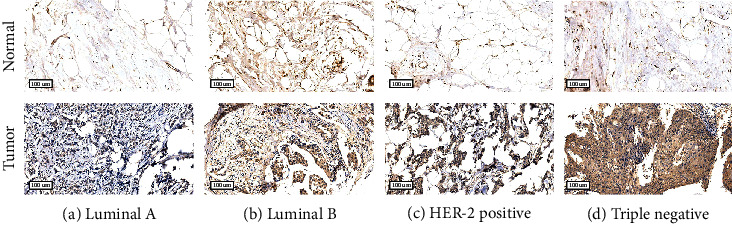
Immunohistochemical staining of RAC2 in different subtypes of BRCA. RAC2 was significantly highly expressed in luminal A, luminal B, Her-2 positive, and triple-negative types.

Finally, to further compare whether RAC2 expression differed in tumor tissues and corresponding normal tissues, we first confirmed from the HPA database that RAC2 was highly expressed in BRCA, COAD, LIHC, LUAD, and PRAD. Then, immunohistochemical staining (IHC) for BRCA was also performed (Figure 13). The IHC results showed that RAC2 was significantly highly expressed in the luminal A, luminal B, Her-2-positive, and triple-negative types (Figure 14).

## 4. Discussion

Previous studies have proved that Rac GTpase is a key signaling component controlling cell adhesion, migration, proliferation, and survival in actin cytoskeletal tissues and mammalian cells [22, 23]. Researches have been increasingly focused on analyzing the role of RAC2 in cancer, and much literature has demonstrated the important role of RAC2 in inhibiting the growth and metastasis of tumor. For example, Xia et al. found that reduced RAC2 expression inhibits osteosarcoma expression through suppressing the Wnt pathway [24]. Liu et al. demonstrated that low expression of RAC2 may reduce the ability of renal clear cell carcinoma cells to proliferate, migrate and invade in vitro, and serve as prognostic biomarkers [25]. In addition, in a study of glioblastoma, Lai et al. revealed that apart from RAC1, RAC2 and RAC3 contribute to the development of glioblastoma [26]. However, the functional relation between RAC2 and tumor and whether it drives or inhibits tumor pathogenesis remained poorly understood. Previous studies limited RAC2 assessment on a few types of cancer, and the role of other tumor types remained elusive. RAC2 in generalized cancer analysis was rarely conducted. Thus, here, we performed a pancancer analysis of RAC2. Our integrated analyses were based on data from TCGA, CPTAC, and GEO databases to explore the molecular characterization and gene expression of RAC2 in 33 different tumors. RAC2 survival prognostic analyses, gene changes, immune infiltration, and enrichment analyses were systematically performed.

After a comprehensive analysis of the differences in RAC2 expression between tumor tissues and control tissues in databases such as TCGA and GTEx, the potential research significance of RAC2 in many cancers was identified. We found that compared with normal tissues, RAC2 expression was significantly elevated in most cancer tissues. Besides, RAC2 expression was deficient in normal tissues and relatively high in immune organs and tissues such as bone marrow, lymph, thymus, tonsil, and various types of immune cells. Due to the overexpression status of RAC2 in pancancer, we next explored its prognostic significance. It was found that RAC2 expression had prognostic value in some cancers, for example, RAC2 affected the overall survival rate of ACC, BRCA, LAML, LGG, SKCM, THYM, and UVM. Especially in ACC, BRCA, and UVM, RAC2 expression affected their OS, DSS, and PFI. As expected, RAC2 may be a biomarker of prognosis in many cancers; however, its prognosis varies across cancers, and these results should be further evaluated from multiple aspects.

From the perspective of immune invasion, we systematically associated RAC2 with immune characteristics in the tumor microenvironment (TME) [27], including immunomodulators, immune checkpoint inhibitors, and tumor-infiltrating immune cells (TIICs). Our results showed a high expression of RAC2 in TME of many cancer types and were significantly positively associated with immunomodulators, immune checkpoint inhibitors, and immune cell infiltration.

MHC/HLAI class expression can be upregulated in immunotherapy, leading to T cell-mediated tumor regression [28, 29]. RAC2 was positively correlated with MHC/HLAI expression; therefore, it may be effective in tumor immunotherapy in inhibiting immune escape. The CXC motif chemokine ligand family in immunomodulators is widely involved in immune cell recruitment and influences tumor progression, such as tumor migration and angiogenesis [30]. Interestingly, based on our previous results, RAC2 expression was increased in ovarian cancer. In survival analysis, low RAC2 expression was associated with poor prognosis, and RAC2 was negatively related to CXCL1. These contradictory results may be due to the fact that the development of tumors was the result of a combination of effects, and analysis of a single molecule was insufficient to elucidate the overall immune role of RAC2 in tumor microenvironment. Immune checkpoint inhibitors are currently the most popular tumor immunotherapy. With the development of high-throughput sequencing technology, an increasing number of immune checkpoints are being identified [30].

We found that RAC2 was positively correlated with many classical immune checkpoint molecules, suggesting that most of these immune checkpoints were closely related to RAC2 expression levels. RAC2 may be a new immune checkpoint. In addition, TMB and MSI showed significant associations with RAC2 in COAD in this study. TMB, which is the total number of somatic nonsynonymous mutations per megabyte (Mut/Mb), is related with the synthesis of abnormal proteins [31, 32]. These proteins can activate antitumor response as a neoantigen [33]. TMB is a useful estimate of tumor-neoantigen burden [34]. MSI is a robust phenotype of mutant caused by DNA mismatch repair defects [34]. It is a potentially important biomarker for predicting ICI response [33, 34]. NEO is a tumor-specific antigen derived from nonsynonymous mutations, which is effective in escaping immune effect, antitumor immune response, and successful cancer immunotherapy. RAC2 was negatively correlated with TMB, MSI, and NEO in ACC and positively correlated with three biomarkers in COAD. This indicated the potential of RAC2 as an immunotherapeutic target for ACC and COAD. Tumor-infiltrating immune cells (TIICs) play an important part in the antitumor mechanism of TME [35]. T cells can destroy tumors and are critical in tumor antigens [36]. Rac2−/−T cells were used to analyze whether Rac2 deletion affected T cell activation by Yu et al. [37]. The results showed that phosphorylation of ERK1/2 and P38 was lower, and antigen-induced calcium 2 + flux was also lower. In addition, RAC2-deficient mice had defects in receptor aggregation during the stimulation of T cell and the differentiation of Th1 [38]. These results suggested that Rac2 deficiency affected transcriptional activation and cytoskeletal recombination during T cell stimulation. NK cells as powerful effector cells of innate immunity can be the first to be detected and can activate immune defense functions. NK cells eliminate transformed cells and protect normal and healthy cells [39]. Activation of GTpaseRac is associated with cytotoxicity mediated by NK cells [40]. However, its role and upstream regulatory factors were still unclear. Tabellini et al. found that germline activation mutations in RAC2 induce PI3K activation [41]. PI3K is a contributing factor in many important aspects of NK cell biology like development, maturation, homing, and function [42]. Therefore, RAC2 may play a tumor-suppressive role in immune cell infiltration. However, studies have also shown that RAC2 also promotes cancer development. Joshi et al. [43]. In vitro and in vivo experiments on Lewis lung cancer, melanoma, pancreatic cancer, and neuroblastoma showed the downstream of a4B1 integrin and MCSF receptors and that RAC2 was activated to inhibit tumor development, metastasis, and macrophage differentiation into the M2 phenotype, thereby promoting tumor growth, angiogenesis, and invasion.

In addition, to further explore the impact of RAC2 on tumor etiology or pathogenesis, we conducted the enrichment analysis on RAC2. A series of enrichment analyses demonstrated that several tumors had a strong association with RAC2. We also observed that “cytokine receptor interaction,” “chemokine signaling pathway,” and “antigen processing and presentation” were enriched in the high expression group of RAC2. This was a validation of the previous studies on immune infiltration, which showed the association of RAC2 with the immune microenvironment.

In summary, RAC2 played an important and bidirectional regulatory role in immune infiltration. This was the first study focusing on the effect of RAC2 in pancancers and provided an innovative perspective on the role of RAC2 in cancer immunotherapy.

However, there were some limitations in this study. Firstly, the study of RAC2 expression in various cancers and its mechanism of action was incomplete. For example, the histology of methylation, phosphorylation, and other modifications has not been studied. The specific role of various parts of the antitumor immune cycle was not explored. Secondly, this paper was based on bioinformatics research, and the results were preliminary and should be studied by conducting more experiments.

## Data Availability

All data generated or analyzed during this study are included in this published article.

## Supplementary Materials

Supplementary 1 Table S1: the top 50 DEGs in the turquoise module. Table S2: *P* values of RAC2 gene with chemokines. Table S3: *P* values of RAC2 gene with receptors. Table S4: *P* values of RAC2 gene with MHCs. Table S5: *P* values of RAC2 gene with immune checkpoint.

Supplementary 2 Figure S1: the 33 tumors in the TGCT database and their corresponding abbreviations. Figure S2: top three tumors with significant correlation between RAC2 and immune score: TGCT, LGG, and KICH. Figure S3: correlation of RAC2 expression and chemokines and receptors. Red indicates a positive correlation, while blue indicates a negative correlation. The dot plot showed the top 4 strongest associations. Figure S4: correlation of RAC2 expression and MHC molecules. Red indicates a positive correlation, while blue indicates a negative correlation. The dot plot showed the top 4 strongest associations. Figure S5-S10: expression of CD4+ T cells, CD8+ T cells, neutrophils, macrophages, dendritic cells, and natural killer cells in relation to individual tumors in each algorithm.

## References

[1] Sung H., Ferlay J., Siegel R. L. (2021). Global cancer statistics 2020: GLOBOCAN estimates of incidence and mortality worldwide for 36 cancers in 185 countries. *CA: a cancer journal for clinicians*.

[2] Mott H. R., Owen D. (2018). Allostery and dynamics in small G proteins. *Biochemical Society transactions*.

[3] Boureux A., Vignal E., Faure S., Fort P. (2007). Evolution of the Rho family of ras-like GTPases in eukaryotes. *Molecular Biology and Evolution*.

[4] Han F., Wang X., Yang Q., Cai M., Wang Z. Y. (2011). Characterization of a RacGTPase up-regulated in the large yellow croaker _Pseudosciaena crocea_ immunity. *Fish & shellfish immunology*.

[5] Didsbury J., Weber R. F., Bokoch G. M., Evans T., Snyderman R. (1989). _rac_ , a novel _ras_ -related family of proteins that are botulinum toxin substrates. *The Journal of biological chemistry*.

[6] Li Z., Wang Q., Peng S. (2020). The metastatic promoter DEPDC1B induces epithelial-mesenchymal transition and promotes prostate cancer cell proliferation via Rac1-PAK1 signaling. *Clinical and translational medicine*.

[7] Mierke C. T., Puder S., Aermes C., Fischer T., Kunschmann T. (2020). Effect of PAK inhibition on cell mechanics depends on Rac1. *Frontiers in cell and developmental biology*.

[8] Li Z., Zhang L., Gao M. (2020). Retraction note: endoplasmic reticulum stress triggers xanthoangelol-induced protective autophagy via activation of JNK/c-Jun axis in hepatocellular carcinoma. *Journal of experimental & clinical cancer research*.

[9] Li Q., Qin T., Bi Z. (2020). Rac1 activates non-oxidative pentose phosphate pathway to induce chemoresistance of breast cancer. *Nature Communications*.

[10] Filippi M. D., Harris C. E., Meller J., Gu Y., Zheng Y., Williams D. A. (2004). Localization of Rac2 via the C terminus and aspartic acid 150 specifies superoxide generation, actin polarity and chemotaxis in neutrophils. *Nature Immunology*.

[11] Sells M. A., Knaus U. G., Bagrodia S., Ambrose D. M., Bokoch G. M., Chernoff J. (1997). Human p21-activated kinase (Pak1) regulates actin organization in mammalian cells. *Current biology*.

[12] Alkhairy O. K., Rezaei N., Graham R. R. (2015). _RAC2_ loss-of-function mutation in 2 siblings with characteristics of common variable immunodeficiency. *The Journal of allergy and clinical immunology*.

[13] Blum A., Wang P., Zenklusen J. C. (2018). SnapShot: TCGA-analyzed tumors. *Cell*.

[14] Charoentong P., Finotello F., Angelova M. (2017). Pancancer immunogenomic analyses reveal genotype-immunophenotype relationships and predictors of response to checkpoint blockade. *Cell Reports*.

[15] Thorsson V., Gibbs D. L., Brown S. D. (2019). The immune landscape of cancer. *Immunity*.

[16] Rizzo A., Ricci A. D., Brandi G. (2021). PD-L1, TMB, MSI, and other predictors of response to immune checkpoint inhibitors in biliary tract cancer. *Cancers*.

[17] Yoshihara K., Shahmoradgoli M., Martínez E. (2013). Inferring tumour purity and stromal and immune cell admixture from expression data. *Nature Communications*.

[18] Langfelder P., Horvath S. (2008). WGCNA: an R package for weighted correlation network analysis. *BMC Bioinformatics*.

[19] Pinto J. P., Kalathur R. K., Oliveira D. V. (2015). StemChecker: a web-based tool to discover and explore stemness signatures in gene sets. *Nucleic acids research*.

[20] Kreso A., Dick J. E. (2014). Evolution of the cancer stem cell model. *Cell Stem Cell*.

[21] Malta T. M., Sokolov A., Gentles A. J. (2018). Machine learning identifies stemness features associated with oncogenic dedifferentiation. *Cell*.

[22] Ridley A. J., Paterson H. F., Johnston C. L., Diekmann D., Hall A. (1992). The small GTP-binding protein rac regulates growth factor-induced membrane ruffling. *Cell*.

[23] del Pozo M. A., Vicente-Manzanares M., Tejedor R., Serrador J. M., Sánchez-Madrid F. (1999). Rho GTPases control migration and polarization of adhesion molecules and cytoskeletal ERM components in T lymphocytes. *European Journal of Immunology*.

[24] Xia P., Gao X., Shao L. (2019). Down-regulation of RAC2 by small interfering RNA restrains the progression of osteosarcoma by suppressing the Wnt signaling pathway. *International journal of biological macromolecules*.

[25] Liu Y., Cheng G., Song Z. (2019). RAC2 acts as a prognostic biomarker and promotes the progression of clear cell renal cell carcinoma. *International journal of oncology*.

[26] Lai Y. J., Tsai J. C., Tseng Y. T. (2017). Small G protein Rac GTPases regulate the maintenance of glioblastoma stem-like cells in vitro and in vivo. *Oncotarget*.

[27] Zhang J., Wang Z., Zhang X. (2022). Large-scale single-cell and bulk sequencing analyses reveal the prognostic value and immune aspects of CD147 in pan-cancer. *Frontiers in immunology*.

[28] Garrido F., Aptsiauri N. (2019). Cancer immune escape: MHC expression in primary tumours versus metastases. *Immunology*.

[29] Dhatchinamoorthy K., Colbert J. D., Rock K. L. (2021). Cancer immune evasion through loss of MHC class I antigen presentation. *Frontiers in immunology*.

[30] Zhang H., Wang Z., Dai Z. (2021). Novel immune infiltrating cell signature based on cell pair algorithm is a prognostic marker in cancer. *Frontiers in immunology*.

[31] Goodman A. M., Kato S., Bazhenova L. (2017). Tumor mutational burden as an independent predictor of response to immunotherapy in diverse cancers. *Molecular cancer therapeutics*.

[32] McNamara M. G., Jacobs T., Lamarca A., Hubner R. A., Valle J. W., Amir E. (2020). Impact of high tumor mutational burden in solid tumors and challenges for biomarker application. *Cancer treatment reviews*.

[33] Yarchoan M., Hopkins A., Jaffee E. M. (2017). Tumor mutational burden and response rate to PD-1 inhibition. *The New England journal of medicine*.

[34] Yamamoto H., Imai K. (2019). An updated review of microsatellite instability in the era of next-generation sequencing and precision medicine. *Seminars in oncology*.

[35] Galli F., Aguilera J. V., Palermo B., Markovic S. N., Nisticò P., Signore A. (2020). Relevance of immune cell and tumor microenvironment imaging in the new era of immunotherapy. *Journal of experimental & clinical cancer research*.

[36] Kishton R. J., Sukumar M., Restifo N. P. (2017). Metabolic regulation of T cell longevity and function in tumor immunotherapy. *Cell metabolism*.

[37] Yu H., Leitenberg D., Li B., Flavell R. A. (2001). Deficiency of small GTPase Rac2 affects T cell activation. *The Journal of experimental medicine*.

[38] Guo F., Cancelas J. A., Hildeman D., Williams D. A., Zheng Y. (2008). Rac GTPase isoforms Rac1 and Rac2 play a redundant and crucial role in T-cell development. *Blood*.

[39] Larsen S. K., Gao Y., Basse P. H. (2014). NK cells in the tumor microenvironment. *Critical reviews in oncogenesis*.

[40] Sakai Y., Tanaka Y., Yanagihara T. (2013). The Rac activator DOCK2 regulates natural killer cell-mediated cytotoxicity in mice through the lytic synapse formation. *Blood*.

[41] Tabellini G., Baronio M., Patrizi O. (2019). The RAC2-PI3K axis regulates human NK cell maturation and function. *Clinical immunology*.

[42] Mace E. M. (2018). Phosphoinositide-3-kinase signaling in human natural killer cells: new insights from primary immunodeficiency. *Frontiers in immunology*.

[43] Joshi S., Singh A. R., Zulcic M. (2014). Rac2 controls tumor growth, metastasis and M1-M2 macrophage differentiation in vivo. *PLoS One*.

