# Supplementary material for: Pancancer Analysis Revealed the Value of RAC2 in Immunotherapy and Cancer Stem Cell
Source: Stem Cells Int. 2023 May 12;2023:8485726. doi: 10.1155/2023/8485726 (PMC10198763; doi:10.1155/2023/8485726)
Supplement: Supplementary 1 — Table S1: the top 50 DEGs in the turquoise module. Table S2: P values of RAC2 gene with chemokines. Table S3: P values of RAC2 gene with receptors. Table S4: P values of RAC2 gene with MHCs. Table S5: P values of RAC2 gene with immune checkpoint. [file 8485726.f1.zip › table S1.pdf]

Gene	Module	MM_R	MM_pvalue
FOXN1	turquoise	0.911484968	0.000622
PAX1	turquoise	0.972492857	1.11E-05
ATRNL1	turquoise	0.910176045	0.000654
ZBED8	turquoise	0.95645577	5.44E-05
S100A14	turquoise	0.882311815	0.001638
PDE7B	turquoise	-0.875256126	0.001994
DSP	turquoise	0.987578091	6.95E-07
CBS	turquoise	0.905579355	0.000776
DUSP10	turquoise	-0.831524515	0.005458
IRF1	turquoise	-0.907099412	0.000734
MMP12	turquoise	-0.922186303	0.000401
UNC5B	turquoise	0.951169862	8.08E-05
GPC4	turquoise	0.889566384	0.001321
ARHGAP42	turquoise	0.923012402	0.000386
REL	turquoise	-0.815838838	0.007333
CROT	turquoise	0.81069546	0.008032
SIX4	turquoise	0.887090771	0.001424
TARBP1	turquoise	0.907169123	0.000732
MTCL1	turquoise	0.930634028	0.00027
SORBS2	turquoise	0.924038036	0.000369
C4B	turquoise	0.949219805	9.24E-05
PKP1	turquoise	0.845956186	0.004049
C4A	turquoise	0.949007061	9.38E-05
CCNL1	turquoise	-0.945591037	0.000117
OXCT1	turquoise	0.831221878	0.005491
DUOX1	turquoise	0.847819948	0.003888
MIR944	turquoise	0.944729457	0.000124
KRT10	turquoise	-0.80059429	0.009534
SPIB	turquoise	-0.887666417	0.001399
MIR4420	turquoise	-0.870024795	0.00229
NRCAM	turquoise	0.843784002	0.004243
PGAP1	turquoise	0.936599339	0.000199
NDRG3	turquoise	0.832302356	0.005375
PCSK6	turquoise	0.802672583	0.009211
C8orf37	turquoise	0.852832613	0.003476
CCDC69	turquoise	-0.905289193	0.000784
PAX9	turquoise	0.9641769	2.77E-05
VPS35	turquoise	0.876199431	0.001944
SRSF7	turquoise	-0.896672809	0.001054
CDS1	turquoise	0.838832346	0.004709
RPGRIP1L	turquoise	0.865747286	0.002554
NPNT	turquoise	0.95805467	4.78E-05
CD83	turquoise	-0.916129112	0.000518
MYO10	turquoise	0.948067176	9.99E-05
DPH6	turquoise	0.916980723	0.0005
DUOXA1	turquoise	0.863748406	0.002684
HSPA4L	turquoise	0.891825951	0.001231
ZNF385D	turquoise	-0.955132279	6.03E-05
MDK	turquoise	0.9534092	6.87E-05
MIR1299	turquoise	0.915280475	0.000536
