# Supplementary material for: Pancancer Analysis Revealed the Value of RAC2 in Immunotherapy and Cancer Stem Cell
Source: Stem Cells Int. 2023 May 12;2023:8485726. doi: 10.1155/2023/8485726 (PMC10198763; doi:10.1155/2023/8485726)
Supplement: Supplementary 1 — Table S1: the top 50 DEGs in the turquoise module. Table S2: P values of RAC2 gene with chemokines. Table S3: P values of RAC2 gene with receptors. Table S4: P values of RAC2 gene with MHCs. Table S5: P values of RAC2 gene with immune checkpoint. [file 8485726.f1.zip › table S3.pdf]

	ACC	BLCA	BRCA	CESC	CHOL	COAD	DLBC	ESCA	GBM	HNSC	KICH	KIRC	KIRP	LAML	LGG	LIHC	LUAD	LUSC	MESO	OV	PAAD	PCPG	PRAD	READ	SARC	SKCM	STAD	TGCT	THCA	THYM	UCEC	UCS	UVM
CCR1	<0.001	<0.001	<0.001	<0.001	<0.001	<0.001	0.068	<0.001	<0.001	<0.001	<0.001	<0.001	<0.001	<0.001	<0.001	<0.001	<0.001	<0.001	<0.001	<0.001	<0.001	<0.001	<0.001	<0.001	<0.001	<0.001	<0.001	<0.001	<0.001	<0.001	<0.001	<0.001	<0.001
CCR2	<0.001	<0.001	<0.001	<0.001	0.017	<0.001	0.118	0.006	<0.001	<0.001	<0.001	<0.001	<0.001	0.003	<0.001	<0.001	<0.001	<0.001	<0.001	<0.001	<0.001	<0.001	<0.001	<0.001	<0.001	<0.001	<0.001	<0.001	<0.001	<0.001	<0.001	<0.001	<0.001
CCR3	0.001	0.614	<0.001	<0.001	0.099	<0.001	0.319	<0.001	<0.001	<0.001	<0.001	<0.001	<0.001	<0.001	<0.001	<0.001	0.193	<0.001	<0.001	<0.001	<0.001	<0.001	<0.001	<0.001	<0.001	<0.001	<0.001	<0.001	<0.001	0.293	0.055	0.008	0.005
CCR4	<0.001	<0.001	<0.001	<0.001	0.010	<0.001	0.150	0.006	<0.001	<0.001	<0.001	<0.001	<0.001	0.571	<0.001	<0.001	<0.001	<0.001	0.001	<0.001	<0.001	<0.001	<0.001	<0.001	<0.001	<0.001	<0.001	<0.001	<0.001	<0.001	<0.001	0.080	<0.001
CCR5	<0.001	<0.001	<0.001	<0.001	0.002	<0.001	0.049	<0.001	<0.001	<0.001	<0.001	<0.001	<0.001	<0.001	<0.001	<0.001	<0.001	<0.001	<0.001	<0.001	<0.001	<0.001	<0.001	<0.001	<0.001	<0.001	<0.001	<0.001	<0.001	<0.001	<0.001	<0.001	<0.001
CCR6	0.234	<0.001	<0.001	<0.001	0.038	<0.001	<0.001	0.287	<0.001	<0.001	<0.001	<0.001	<0.001	0.038	<0.001	<0.001	<0.001	<0.001	0.003	<0.001	<0.001	<0.001	<0.001	0.019	<0.001	<0.001	<0.001	<0.001	<0.001	<0.001	<0.001	0.362	<0.001
CCR7	<0.001	0.763	<0.001	<0.001	0.009	<0.001	0.012	0.039	<0.001	<0.001	<0.001	<0.001	<0.001	0.206	<0.001	<0.001	<0.001	<0.001	<0.001	<0.001	<0.001	<0.001	<0.001	<0.001	<0.001	<0.001	<0.001	<0.001	<0.001	<0.001	<0.001	0.005	<0.001
CCR8	<0.001	<0.001	<0.001	<0.001	0.125	<0.001	0.149	<0.001	<0.001	<0.001	<0.001	<0.001	<0.001	0.207	<0.001	<0.001	<0.001	<0.001	<0.001	<0.001	<0.001	<0.001	<0.001	<0.001	<0.001	<0.001	<0.001	0.022	<0.001	<0.001	<0.001	0.061	<0.001
CCR9	0.017	0.383	<0.001	0.092	0.465	0.002	0.030	0.981	0.050	0.005	0.408	<0.001	0.292	0.045	0.005	<0.001	<0.001	<0.001	0.149	<0.001	<0.001	<0.001	<0.001	0.106	0.010	<0.001	<0.001	<0.001	<0.001	<0.001	0.087	0.478	0.031
CCR10	0.610	0.615	<0.001	0.542	0.666	<0.001	0.194	0.160	0.476	0.176	0.119	0.017	<0.001	0.036	<0.001	<0.001	0.191	<0.001	0.808	0.849	0.007	0.063	<0.001	<0.001	<0.001	<0.001	<0.001	0.788	<0.001	0.418	<0.001	0.598	<0.001
CXCR1	0.349	<0.001	<0.001	0.007	0.025	<0.001	0.782	0.009	<0.001	<0.001	<0.001	<0.001	<0.001	<0.001	<0.001	<0.001	0.002	<0.001	0.326	<0.001	0.014	<0.001	<0.001	<0.001	<0.001	<0.001	<0.001	0.239	<0.001	0.006	<0.001	0.051	0.157
CXCR2	<0.001	0.012	<0.001	<0.001	0.037	<0.001	0.299	0.989	<0.001	0.918	<0.001	<0.001	<0.001	<0.001	<0.001	<0.001	<0.001	<0.001	0.059	<0.001	0.002	<0.001	<0.001	<0.001	<0.001	<0.001	0.001	0.011	<0.001	0.481	<0.001	0.029	0.015
CXCR3	<0.001	<0.001	<0.001	<0.001	0.004	<0.001	0.047	0.023	<0.001	<0.001	<0.001	<0.001	<0.001	0.020	<0.001	<0.001	<0.001	<0.001	<0.001	<0.001	<0.001	<0.001	<0.001	0.067	<0.001	<0.001	<0.001	<0.001	<0.001	0.602	<0.001	<0.001	<0.001
CXCR4	0.015	<0.001	<0.001	0.064	0.001	<0.001	0.085	0.062	<0.001	<0.001	<0.001	<0.001	<0.001	0.001	<0.001	<0.001	<0.001	<0.001	<0.001	0.133	<0.001	<0.001	<0.001	<0.001	<0.001	<0.001	<0.001	<0.001	<0.001	<0.001	0.004	0.293	0.008
CXCR5	0.314	<0.001	<0.001	<0.001	0.108	<0.001	0.006	0.020	0.055	<0.001	<0.001	<0.001	<0.001	0.187	<0.001	<0.001	<0.001	<0.001	0.007	<0.001	<0.001	<0.001	<0.001	<0.001	0.019	<0.001	<0.001	<0.001	<0.001	0.021	<0.001	0.029	<0.001
CXCR6	<0.001	<0.001	<0.001	<0.001	0.003	<0.001	0.093	<0.001	<0.001	<0.001	<0.001	<0.001	<0.001	0.149	<0.001	<0.001	<0.001	<0.001	<0.001	<0.001	<0.001	<0.001	<0.001	<0.001	<0.001	<0.001	<0.001	<0.001	<0.001	<0.001	<0.001	0.002	<0.001
XCR1	<0.001	0.715	<0.001	<0.001	0.032	<0.001	0.008	0.049	<0.001	0.003	<0.001	<0.001	<0.001	0.634	<0.001	<0.001	<0.001	<0.001	<0.001	<0.001	<0.001	<0.001	<0.001	<0.001	<0.001	<0.001	<0.001	<0.001	<0.001	<0.001	<0.001	<0.001	<0.001
CX3CR1	0.002	<0.001	<0.001	<0.001	0.423	<0.001	0.107	0.003	<0.001	<0.001	<0.001	<0.001	<0.001	<0.001	<0.001	<0.001	<0.001	<0.001	0.557	<0.001	0.132	0.003	<0.001	<0.001	<0.001	<0.001	<0.001	0.023	<0.001	0.003	<0.001	0.908	0.218
