# Supplementary material for: Pancancer Analysis Revealed the Value of RAC2 in Immunotherapy and Cancer Stem Cell
Source: Stem Cells Int. 2023 May 12;2023:8485726. doi: 10.1155/2023/8485726 (PMC10198763; doi:10.1155/2023/8485726)
Supplement: Supplementary 1 — Table S1: the top 50 DEGs in the turquoise module. Table S2: P values of RAC2 gene with chemokines. Table S3: P values of RAC2 gene with receptors. Table S4: P values of RAC2 gene with MHCs. Table S5: P values of RAC2 gene with immune checkpoint. [file 8485726.f1.zip › table S4.pdf]

	ACC	BLCA	BRCA	CESC	CHOL	COAD	DLBC	ESCA	GBM	HNSC	KICH	KIRC	KIRP	LAML	LGG	LIHC	LUAD
B2M	<0.001	<0.001	<0.001	<0.001	0.087	<0.001	0.023	<0.001	<0.001	<0.001	0.002	<0.001	<0.001	0.280	<0.001	<0.001	<0.001
HLA-A	<0.001	<0.001	<0.001	<0.001	0.167	<0.001	0.831	<0.001	<0.001	<0.001	<0.001	<0.001	<0.001	<0.001	<0.001	<0.001	<0.001
HLA-B	<0.001	<0.001	<0.001	<0.001	0.123	<0.001	0.861	<0.001	<0.001	<0.001	<0.001	<0.001	<0.001	<0.001	<0.001	<0.001	<0.001
HLA-C	<0.001	<0.001	<0.001	<0.001	0.249	<0.001	0.729	0.002	<0.001	<0.001	0.001	<0.001	<0.001	<0.001	<0.001	<0.001	<0.001
HLA-E	<0.001	<0.001	<0.001	<0.001	0.038	<0.001	0.032	<0.001	<0.001	<0.001	<0.001	<0.001	<0.001	<0.001	<0.001	<0.001	<0.001
HLA-F	<0.001	<0.001	<0.001	<0.001	0.126	<0.001	0.308	<0.001	<0.001	<0.001	<0.001	<0.001	<0.001	<0.001	<0.001	<0.001	<0.001
HLA-G	0.135	<0.001	<0.001	<0.001	0.186	<0.001	0.194	0.007	<0.001	<0.001	0.024	<0.001	<0.001	0.010	<0.001	<0.001	<0.001
HLA-H	0.064	<0.001	<0.001	<0.001	0.039	<0.001	0.402	0.108	0.001	<0.001	0.005	<0.001	<0.001	<0.001	<0.001	<0.001	<0.001
HLA-J	0.166	<0.001	<0.001	<0.001	0.916	0.001	0.522	0.269	<0.001	<0.001	0.001	<0.001	<0.001	0.764	<0.001	<0.001	<0.001
HLA-K	0.134	<0.001	<0.001	0.002	0.538	<0.001	0.137	0.472	0.821	0.288	0.058	<0.001	<0.001	0.002	<0.001	0.001	<0.001
HLA-L	0.689	<0.001	<0.001	<0.001	0.645	<0.001	0.044	0.109	<0.001	<0.001	0.062	<0.001	<0.001	0.032	<0.001	<0.001	<0.001
HLA-DRA	<0.001	<0.001	<0.001	<0.001	0.006	<0.001	0.082	<0.001	<0.001	<0.001	<0.001	<0.001	<0.001	<0.001	<0.001	<0.001	<0.001
HLA-DRB1	<0.001	<0.001	<0.001	<0.001	0.011	<0.001	0.642	<0.001	<0.001	<0.001	<0.001	<0.001	<0.001	<0.001	<0.001	<0.001	<0.001
HLA-DRB5	<0.001	<0.001	<0.001	<0.001	0.185	<0.001	0.132	<0.001	<0.001	<0.001	<0.001	<0.001	<0.001	<0.001	<0.001	<0.001	<0.001
HLA-DRB6	<0.001	<0.001	<0.001	<0.001	0.054	<0.001	0.619	0.038	<0.001	<0.001	<0.001	<0.001	<0.001	<0.001	<0.001	<0.001	<0.001
HLA-DRB9	0.001	<0.001	<0.001	<0.001	0.044	<0.001	0.365	<0.001	<0.001	<0.001	0.001	<0.001	<0.001	0.002	<0.001	<0.001	<0.001
HLA-DQA1	<0.001	<0.001	<0.001	<0.001	<0.001	<0.001	0.064	<0.001	<0.001	<0.001	<0.001	<0.001	<0.001	0.001	<0.001	<0.001	<0.001
HLA-DQB1	<0.001	<0.001	<0.001	<0.001	<0.001	<0.001	0.463	<0.001	<0.001	<0.001	<0.001	<0.001	<0.001	<0.001	<0.001	<0.001	<0.001
HLA-DQA2	<0.001	<0.001	<0.001	<0.001	0.021	<0.001	0.251	<0.001	0.005	<0.001	<0.001	<0.001	<0.001	0.112	<0.001	<0.001	<0.001
HLA-DQB2	<0.001	<0.001	<0.001	<0.001	0.004	<0.001	0.299	<0.001	<0.001	<0.001	<0.001	<0.001	<0.001	0.004	<0.001	<0.001	<0.001
HLA-DQB3		0.701	0.379	0.472	0.092	0.004	0.781	0.159	0.579	0.327	0.526	0.455	0.992	0.126	0.503	0.355	0.542
HLA-DPA1	<0.001	<0.001	<0.001	<0.001	<0.001	<0.001	0.117	<0.001	<0.001	<0.001	<0.001	<0.001	<0.001	<0.001	<0.001	<0.001	<0.001
HLA-DPB1	<0.001	<0.001	<0.001	<0.001	0.022	<0.001	0.595	<0.001	<0.001	<0.001	<0.001	<0.001	<0.001	<0.001	<0.001	<0.001	<0.001
HLA-DPA2	0.050	0.002	<0.001	0.296	0.115	0.025	0.033	0.517	0.329	<0.001	0.035	<0.001	0.023	0.995	0.012	<0.001	<0.001
HLA-DPB2	<0.001	<0.001	<0.001	<0.001	0.332	<0.001	0.073	<0.001	<0.001	<0.001	<0.001	<0.001	<0.001	0.008	<0.001	<0.001	<0.001
TAP1	0.014	<0.001	<0.001	<0.001	0.011	<0.001	0.069	<0.001	0.048	<0.001	<0.001	<0.001	<0.001	<0.001	<0.001	<0.001	<0.001
TAP2	0.311	<0.001	<0.001	<0.001	0.017	<0.001	0.037	<0.001	0.039	<0.001	<0.001	<0.001	<0.001	0.002	<0.001	<0.001	<0.001
TAPBP	0.020	<0.001	<0.001	<0.001	0.165	<0.001	0.475	<0.001	<0.001	<0.001	<0.001	<0.001	<0.001	<0.001	<0.001	<0.001	<0.001

	LUSC	MESO	OV	PAAD	PCPG	PRAD	READ	SARC	SKCM	STAD	TGCT	THCA	THYM	UCEC	UCS	UVM
B2M	<0.001	<0.001	<0.001	<0.001	<0.001	<0.001	<0.001	<0.001	<0.001	<0.001	<0.001	<0.001	0.049	<0.001	<0.001	<0.001
HLA-A	<0.001	0.003	<0.001	<0.001	<0.001	<0.001	<0.001	<0.001	<0.001	<0.001	<0.001	<0.001	0.002	<0.001	<0.001	<0.001
HLA-B	<0.001	0.001	<0.001	<0.001	<0.001	<0.001	<0.001	<0.001	<0.001	<0.001	<0.001	<0.001	<0.001	<0.001	<0.001	<0.001
HLA-C	<0.001	<0.001	<0.001	<0.001	<0.001	<0.001	<0.001	<0.001	<0.001	<0.001	<0.001	<0.001	<0.001	<0.001	<0.001	<0.001
HLA-E	<0.001	0.007	<0.001	<0.001	<0.001	<0.001	<0.001	<0.001	<0.001	<0.001	<0.001	<0.001	0.009	<0.001	<0.001	<0.001
HLA-F	<0.001	0.003	<0.001	<0.001	<0.001	<0.001	<0.001	<0.001	<0.001	<0.001	<0.001	<0.001	0.024	<0.001	<0.001	<0.001
HLA-G	<0.001	0.016	<0.001	0.020	<0.001	<0.001	<0.001	<0.001	<0.001	<0.001	0.001	<0.001	0.001	<0.001	<0.001	<0.001
HLA-H	<0.001	<0.001	<0.001	<0.001	<0.001	<0.001	0.036	<0.001	<0.001	<0.001	<0.001	<0.001	<0.001	<0.001	<0.001	<0.001
HLA-J	<0.001	0.013	<0.001	0.069	<0.001	<0.001	0.733	<0.001	<0.001	<0.001	<0.001	<0.001	<0.001	<0.001	<0.001	<0.001
HLA-K	0.001	0.117	0.006	0.069	<0.001	<0.001	0.067	0.006	<0.001	0.129	0.063	<0.001	0.232	<0.001	0.079	0.430
HLA-L	<0.001	0.018	<0.001	<0.001	<0.001	<0.001	0.001	<0.001	<0.001	<0.001	<0.001	<0.001	0.008	<0.001	<0.001	0.003
HLA-DRA	<0.001	<0.001	<0.001	<0.001	<0.001	<0.001	<0.001	<0.001	<0.001	<0.001	<0.001	<0.001	0.002	<0.001	<0.001	<0.001
HLA-DRB1	<0.001	<0.001	<0.001	<0.001	<0.001	<0.001	<0.001	<0.001	<0.001	<0.001	<0.001	<0.001	0.064	<0.001	<0.001	<0.001
HLA-DRB5	<0.001	<0.001	<0.001	<0.001	<0.001	<0.001	<0.001	<0.001	<0.001	<0.001	<0.001	<0.001	0.037	<0.001	0.005	<0.001
HLA-DRB6	<0.001	<0.001	<0.001	<0.001	<0.001	<0.001	<0.001	<0.001	<0.001	<0.001	<0.001	<0.001	0.553	<0.001	<0.001	<0.001
HLA-DRB9	<0.001	<0.001	<0.001	<0.001	<0.001	<0.001	<0.001	<0.001	<0.001	<0.001	<0.001	<0.001	0.008	<0.001	0.015	<0.001
HLA-DQA1	<0.001	<0.001	<0.001	<0.001	<0.001	<0.001	<0.001	<0.001	<0.001	<0.001	<0.001	<0.001	0.109	<0.001	<0.001	<0.001
HLA-DQB1	<0.001	<0.001	<0.001	<0.001	<0.001	<0.001	<0.001	<0.001	<0.001	<0.001	<0.001	<0.001	0.996	<0.001	<0.001	<0.001
HLA-DQA2	<0.001	<0.001	<0.001	<0.001	<0.001	<0.001	0.002	<0.001	<0.001	<0.001	<0.001	<0.001	0.677	<0.001	0.144	<0.001
HLA-DQB2	<0.001	<0.001	<0.001	<0.001	0.007	<0.001	<0.001	<0.001	<0.001	<0.001	<0.001	<0.001	0.649	<0.001	0.082	<0.001
HLA-DQB3	0.859	0.543	0.993	0.589		0.566	0.291	0.121	0.475	0.372	0.663	0.133	0.304	0.740		0.683
HLA-DPA1	<0.001	<0.001	<0.001	<0.001	<0.001	<0.001	<0.001	<0.001	<0.001	<0.001	<0.001	<0.001	0.010	<0.001	<0.001	<0.001
HLA-DPB1	<0.001	<0.001	<0.001	<0.001	<0.001	<0.001	<0.001	<0.001	<0.001	<0.001	<0.001	<0.001	0.671	<0.001	<0.001	<0.001
HLA-DPA2	<0.001	0.130	<0.001	<0.001	0.513	0.002	0.042	0.430	<0.001	<0.001	<0.001	<0.001	0.969	0.052	0.413	0.008
HLA-DPB2	<0.001	0.049	<0.001	<0.001	0.001	<0.001	0.001	<0.001	<0.001	<0.001	0.182	<0.001	0.547	<0.001	<0.001	<0.001
TAP1	<0.001	0.004	<0.001	<0.001	<0.001	<0.001	<0.001	<0.001	<0.001	<0.001	<0.001	<0.001	0.058	<0.001	<0.001	<0.001
TAP2	<0.001	<0.001	<0.001	<0.001	<0.001	<0.001	0.339	<0.001	<0.001	<0.001	<0.001	<0.001	0.048	<0.001	0.001	<0.001
TAPBP	<0.001	0.062	<0.001	<0.001	<0.001	<0.001	0.050	<0.001	<0.001	<0.001	<0.001	<0.001	0.011	<0.001	<0.001	<0.001
