# Supplementary material for: Pancancer Analysis Revealed the Value of RAC2 in Immunotherapy and Cancer Stem Cell
Source: Stem Cells Int. 2023 May 12;2023:8485726. doi: 10.1155/2023/8485726 (PMC10198763; doi:10.1155/2023/8485726)
Supplement: Supplementary 1 — Table S1: the top 50 DEGs in the turquoise module. Table S2: P values of RAC2 gene with chemokines. Table S3: P values of RAC2 gene with receptors. Table S4: P values of RAC2 gene with MHCs. Table S5: P values of RAC2 gene with immune checkpoint. [file 8485726.f1.zip › table S5.pdf]

	ACC	BLCA	BRCA	CESC	CHOL	COAD	DLBC	ESCA	GBM	HNSC	KICH	KIRC	KIRP	LAML	LGG	LIHC	LUAD	LUSC	MESO	OV	PAAD	PCPG	PRAD	READ	SARC	SKCM	STAD	TGCT	THCA	THYM	UCEC	UCS	UVM
ADORA2A	0.473	<0.001	<0.001	0.107	0.107	<0.001	0.009	0.572	0.957	<0.001	0.002	0.010	<0.001	0.097	0.995	<0.001	<0.001	<0.001	<0.001	<0.001	<0.001	<0.001	<0.001	0.007	0.568	<0.001	<0.001	0.966	<0.001	<0.001	<0.001	0.775	0.217
BTLA	0.112	<0.001	<0.001	<0.001	<0.001	0.002	<0.001	0.002	0.118	<0.001	0.002	<0.001	<0.001	0.886	<0.001	<0.001	<0.001	<0.001	<0.001	0.002	<0.001	<0.001	<0.001	<0.001	<0.001	<0.001	<0.001	<0.001	<0.001	<0.001	<0.001	0.007	<0.001
CD160	0.148	<0.001	<0.001	<0.001	<0.001	<0.001	0.002	0.436	0.163	<0.001	<0.001	<0.001	<0.001	0.128	<0.001	<0.001	<0.001	<0.001	0.683	<0.001	0.138	<0.001	<0.001	0.510	0.010	<0.001	<0.001	<0.001	<0.001	<0.001	<0.001	0.855	<0.001
CD244	<0.001	<0.001	<0.001	<0.001	<0.001	0.012	0.039	<0.001	<0.001	<0.001	<0.001	<0.001	<0.001	0.006	<0.001	<0.001	<0.001	<0.001	0.172	<0.001	<0.001	<0.001	<0.001	<0.001	<0.001	<0.001	<0.001	<0.001	<0.001	<0.001	<0.001	0.001	0.002
CD274	<0.001	<0.001	<0.001	<0.001	<0.001	<0.001	0.065	<0.001	<0.001	<0.001	0.227	<0.001	0.138	0.250	<0.001	<0.001	<0.001	<0.001	<0.001	<0.001	<0.001	<0.001	<0.001	<0.001	<0.001	<0.001	<0.001	<0.001	<0.001	0.019	<0.001	0.004	<0.001
CD96	0.002	0.006	<0.001	<0.001	<0.001	<0.001	0.021	<0.001	<0.001	<0.001	<0.001	<0.001	<0.001	0.376	<0.001	<0.001	<0.001	<0.001	<0.001	<0.001	<0.001	<0.001	<0.001	<0.001	<0.001	<0.001	<0.001	<0.001	<0.001	<0.001	<0.001	0.001	<0.001
CSF1R	<0.001	<0.001	<0.001	<0.001	<0.001	<0.001	0.115	<0.001	<0.001	<0.001	<0.001	<0.001	<0.001	<0.001	<0.001	<0.001	<0.001	<0.001	<0.001	<0.001	<0.001	<0.001	<0.001	<0.001	<0.001	<0.001	<0.001	<0.001	<0.001	<0.001	<0.001	<0.001	<0.001
CTLA4	<0.001	<0.001	<0.001	<0.001	<0.001	<0.001	0.202	<0.001	<0.001	<0.001	<0.001	<0.001	<0.001	0.274	<0.001	<0.001	<0.001	<0.001	<0.001	<0.001	<0.001	<0.001	<0.001	<0.001	<0.001	<0.001	<0.001	<0.001	<0.001	<0.001	<0.001	0.006	<0.001
HAVCR2	<0.001	<0.001	<0.001	<0.001	<0.001	<0.001	0.640	<0.001	<0.001	<0.001	<0.001	<0.001	0.009	<0.001	<0.001	<0.001	<0.001	<0.001	<0.001	<0.001	<0.001	<0.001	<0.001	<0.001	<0.001	<0.001	<0.001	<0.001	<0.001	<0.001	<0.001	<0.001	<0.001
IDO1	0.646	<0.001	<0.001	<0.001	<0.001	<0.001	0.349	<0.001	<0.001	<0.001	0.580	<0.001	0.011	0.048	<0.001	<0.001	<0.001	<0.001	<0.001	<0.001	<0.001	<0.001	<0.001	<0.001	<0.001	<0.001	<0.001	<0.001	<0.001	0.541	<0.001	0.015	<0.001
IL10	<0.001	<0.001	<0.001	<0.001	<0.001	0.165	0.022	<0.001	<0.001	<0.001	<0.001	<0.001	<0.001	0.002	<0.001	<0.001	<0.001	<0.001	0.001	<0.001	<0.001	<0.001	<0.001	<0.001	<0.001	<0.001	<0.001	<0.001	<0.001	0.844	<0.001	0.002	<0.001
IL10RB	0.785	<0.001	<0.001	0.258	0.258	<0.001	0.691	0.091	<0.001	<0.001	<0.001	<0.001	<0.001	<0.001	<0.001	<0.001	<0.001	<0.001	0.157	<0.001	<0.001	<0.001	<0.001	0.659	<0.001	<0.001	0.021	<0.001	<0.001	<0.001	<0.001	0.011	<0.001
KDR	0.135	0.931	<0.001	0.361	0.361	<0.001	0.007	0.415	0.696	<0.001	0.002	0.826	0.041	0.009	0.016	0.102	0.017	<0.001	0.413	<0.001	0.025	<0.001	<0.001	0.214	0.474	0.001	<0.001	<0.001	<0.001	<0.001	0.020	0.228	<0.001
KIR2DL1	0.003	<0.001	<0.001	<0.001	<0.001	<0.001	0.334	<0.001	0.033	<0.001	<0.001	<0.001	<0.001	0.247	0.007	<0.001	<0.001	<0.001	0.287	<0.001	0.090	<0.001	<0.001	0.484	<0.001	<0.001	<0.001	<0.001	<0.001	0.023	<0.001	0.753	0.006
KIR2DL3	0.044	<0.001	<0.001	<0.001	<0.001	0.005	0.208	<0.001	0.047	<0.001	0.004	<0.001	<0.001	0.025	<0.001	<0.001	<0.001	<0.001	0.322	<0.001	0.069	<0.001	<0.001	0.202	<0.001	<0.001	<0.001	<0.001	<0.001	0.021	<0.001	0.529	<0.001
LAG3	0.418	<0.001	<0.001	<0.001	<0.001	0.017	0.150	<0.001	0.083	<0.001	<0.001	<0.001	<0.001	0.047	<0.001	<0.001	<0.001	<0.001	0.002	<0.001	<0.001	<0.001	<0.001	<0.001	<0.001	<0.001	<0.001	<0.001	<0.001	<0.001	<0.001	<0.001	<0.001
LGALS9	<0.001	<0.001	<0.001	0.060	0.060	<0.001	0.865	0.836	<0.001	<0.001	<0.001	<0.001	<0.001	<0.001	<0.001	<0.001	<0.001	<0.001	<0.001	<0.001	<0.001	<0.001	<0.001	<0.001	<0.001	<0.001	<0.001	<0.001	<0.001	<0.001	<0.001	<0.001	<0.001
PDCD1	<0.001	<0.001	<0.001	<0.001	<0.001	0.858	0.010	<0.001	<0.001	<0.001	<0.001	<0.001	<0.001	<0.001	<0.001	<0.001	<0.001	<0.001	<0.001	<0.001	<0.001	<0.001	<0.001	<0.001	<0.001	<0.001	<0.001	<0.001	<0.001	<0.001	<0.001	0.006	<0.001
PDCD1LG2	<0.001	<0.001	<0.001	<0.001	<0.001	<0.001	0.335	<0.001	<0.001	<0.001	<0.001	<0.001	<0.001	0.354	<0.001	<0.001	<0.001	<0.001	<0.001	<0.001	<0.001	<0.001	<0.001	<0.001	<0.001	<0.001	<0.001	<0.001	<0.001	0.435	<0.001	0.004	0.002
TGFB1	0.024	<0.001	<0.001	<0.001	<0.001	<0.001	0.080	<0.001	<0.001	<0.001	<0.001	<0.001	<0.001	<0.001	<0.001	<0.001	<0.001	<0.001	<0.001	<0.001	<0.001	<0.001	<0.001	<0.001	<0.001	<0.001	<0.001	<0.001	<0.001	0.205	<0.001	<0.001	0.002
TGFBR1	0.262	<0.001	0.843	0.014	0.014	<0.001	<0.001	<0.001	<0.001	<0.001	0.124	<0.001	<0.001	0.857	<0.001	<0.001	0.172	0.006	0.130	0.007	0.255	<0.001	<0.001	0.785	0.117	0.010	0.738	0.594	<0.001	<0.001	0.153	0.223	0.084
TIGIT	<0.001	<0.001	<0.001	<0.001	<0.001	<0.001	0.210	<0.001	<0.001	<0.001	<0.001	<0.001	<0.001	0.090	<0.001	<0.001	<0.001	<0.001	<0.001	<0.001	<0.001	<0.001	<0.001	<0.001	<0.001	<0.001	<0.001	<0.001	<0.001	<0.001	<0.001	<0.001	<0.001
VTCN1	0.584	<0.001	0.538	<0.001	<0.001	<0.001	0.419	0.749	0.281	0.866	0.026	0.381	0.004	0.581	0.500	<0.001	0.889	<0.001	0.069	<0.001	0.584	0.866	<0.001	0.083	<0.001	<0.001	0.298	<0.001	<0.001	0.008	0.526	0.378	0.210
ARG1	0.935	0.004	0.476	<0.001	<0.001	<0.001	0.313	0.186	0.136	<0.001	0.048	0.019	0.001	0.909	0.444	0.007	0.012	<0.001	0.925	0.392	0.721	0.024	0.119	0.617	0.012	0.778	0.423	0.016	0.029	0.276	0.940	0.208	0.194
CD276	0.313	<0.001	0.043	0.057	0.057	<0.001	0.149	<0.001	0.002	<0.001	0.001	<0.001	<0.001	0.087	<0.001	<0.001	<0.001	0.009	0.787	0.002	<0.001	<0.001	0.036	<0.001	<0.001	0.045	0.134	<0.001	<0.001	<0.001	0.901	0.649	0.007
EDNRB	0.696	0.030	<0.001	0.183	0.183	<0.001	0.027	0.576	<0.001	0.013	0.229	0.016	0.091	0.135	0.009	0.138	0.081	<0.001	0.047	0.426	0.006	<0.001	<0.001	0.214	0.140	<0.001	<0.001	0.046	<0.001	<0.001	0.710	0.312	<0.001
IL12A	<0.001	<0.001	<0.001	0.507	0.507	<0.001	0.003	0.061	<0.001	<0.001	<0.001	<0.001	<0.001	0.836	0.036	<0.001	<0.001	0.981	<0.001	<0.001	<0.001	0.350	<0.001	0.026	0.004	<0.001	<0.001	0.839	<0.001	<0.001	0.176	0.394	0.008
IL13	<0.001	<0.001	<0.001	<0.001	<0.001	0.144	0.583	0.588	<0.001	<0.001	0.004	0.042	0.045	0.524	0.141	0.022	<0.001	<0.001	0.411	<0.001	0.121	0.176	0.004	0.003	0.419	0.007	0.002	0.972	0.002	0.270	0.006	0.773	0.030
IL4	0.451	0.115	0.009	0.760	0.760	<0.001	0.408	0.938	0.491	0.003	0.009	0.196	<0.001	0.706	0.194	<0.001	0.984	<0.001	0.093	0.288	0.675	0.046	0.614	0.460	0.339	0.520	0.075	<0.001	<0.001	<0.001	0.513	0.756	0.913
SLAMF7	<0.001	<0.001	<0.001	<0.001	<0.001	0.005	0.168	<0.001	<0.001	<0.001	<0.001	<0.001	<0.001	0.762	<0.001	<0.001	<0.001	<0.001	<0.001	<0.001	<0.001	<0.001	<0.001	<0.001	<0.001	<0.001	<0.001	<0.001	<0.001	0.102	<0.001	0.006	<0.001
VEGFA	0.004	0.002	<0.001	0.069	0.069	<0.001	0.220	0.314	0.999	0.188	0.917	0.535	0.880	0.010	<0.001	0.022	0.329	0.022	0.426	0.200	0.102	<0.001	0.959	0.284	0.007	0.262	0.315	<0.001	<0.001	<0.001	0.014	0.773	0.052
VEGFB	0.527	<0.001	<0.001	0.003	0.003	<0.001	0.026	<0.001	0.583	0.051	0.467	0.609	0.095	0.004	<0.001	<0.001	0.331	0.022	0.006	0.581	<0.001	0.144	0.061	0.011	0.069	<0.001	0.016	0.820	<0.001	0.823	0.594	0.096	0.814
