# Supplementary figures and images for: Pancancer Analysis Revealed the Value of RAC2 in Immunotherapy and Cancer Stem Cell
Source: Stem Cells Int. 2023 May 12;2023:8485726. doi: 10.1155/2023/8485726 (PMC10198763; doi:10.1155/2023/8485726)

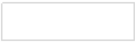

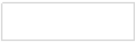

Supplement: Supplementary 1 — Table S1: the top 50 DEGs in the turquoise module. Table S2: P values of RAC2 gene with chemokines. Table S3: P values of RAC2 gene with receptors. Table S4: P values of RAC2 gene with MHCs. Table S5: P values of RAC2 gene with immune checkpoint. [file 8485726.f1.zip › table S2.pdf]

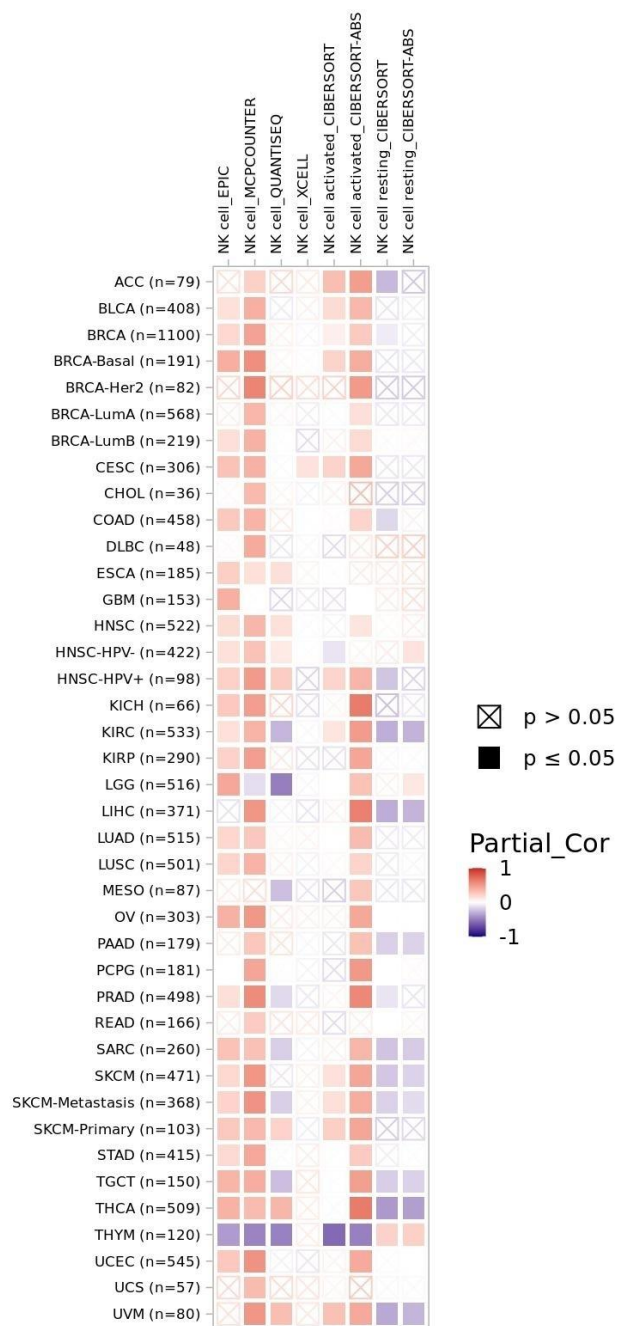

Supplement: Supplementary 2 — Figure S1: the 33 tumors in the TGCT database and their corresponding abbreviations. Figure S2: top three tumors with significant correlation between RAC2 and immune score: TGCT, LGG, and KICH. Figure S3: correlation of RAC2 expression and chemokines and receptors. Red indicates a positive correlation, while blue indicates a negative correlation. The dot plot showed the top 4 strongest associations. Figure S4: correlation of RAC2 expression and MHC molecules. Red indicates a positive correlation, while blue indicates a negative correlation. The dot plot showed the top 4 strongest associations. Figure S5-S10: expression of CD4+ T cells, CD8+ T cells, neutrophils, macrophages, dendritic cells, and natural killer cells in relation to individual tumors in each algorithm. [file 8485726.f2.zip › Figure S10.pdf]

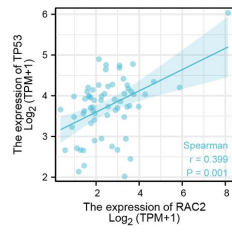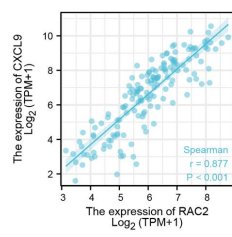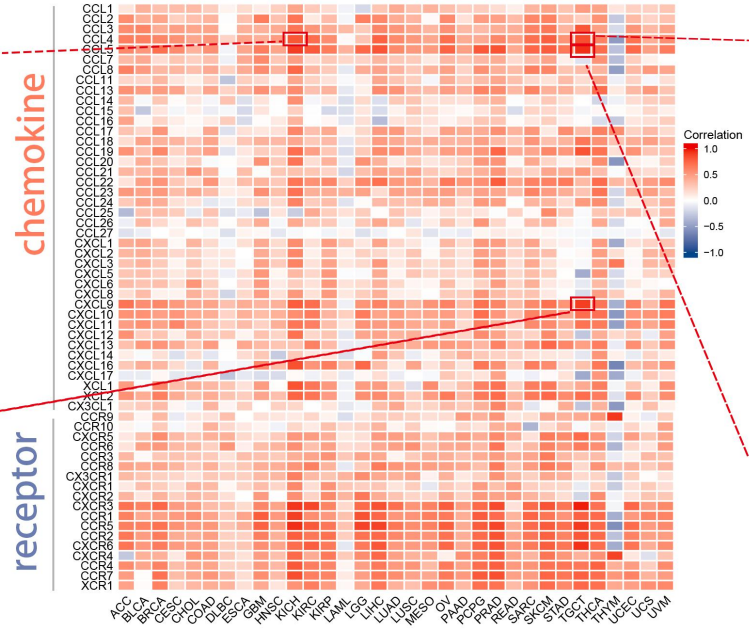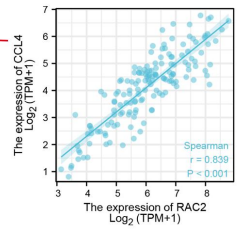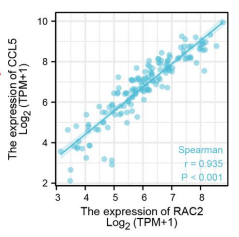

Supplement: Supplementary 2 — Figure S1: the 33 tumors in the TGCT database and their corresponding abbreviations. Figure S2: top three tumors with significant correlation between RAC2 and immune score: TGCT, LGG, and KICH. Figure S3: correlation of RAC2 expression and chemokines and receptors. Red indicates a positive correlation, while blue indicates a negative correlation. The dot plot showed the top 4 strongest associations. Figure S4: correlation of RAC2 expression and MHC molecules. Red indicates a positive correlation, while blue indicates a negative correlation. The dot plot showed the top 4 strongest associations. Figure S5-S10: expression of CD4+ T cells, CD8+ T cells, neutrophils, macrophages, dendritic cells, and natural killer cells in relation to individual tumors in each algorithm. [file 8485726.f2.zip › Figure S2 (1).pdf]

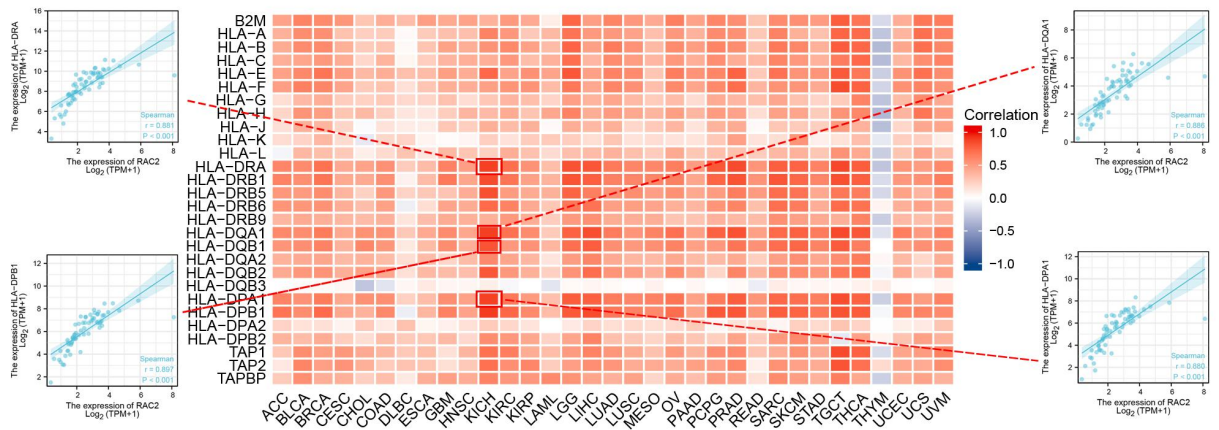

Supplement: Supplementary 2 — Figure S1: the 33 tumors in the TGCT database and their corresponding abbreviations. Figure S2: top three tumors with significant correlation between RAC2 and immune score: TGCT, LGG, and KICH. Figure S3: correlation of RAC2 expression and chemokines and receptors. Red indicates a positive correlation, while blue indicates a negative correlation. The dot plot showed the top 4 strongest associations. Figure S4: correlation of RAC2 expression and MHC molecules. Red indicates a positive correlation, while blue indicates a negative correlation. The dot plot showed the top 4 strongest associations. Figure S5-S10: expression of CD4+ T cells, CD8+ T cells, neutrophils, macrophages, dendritic cells, and natural killer cells in relation to individual tumors in each algorithm. [file 8485726.f2.zip › Figure S3 (1).pdf]

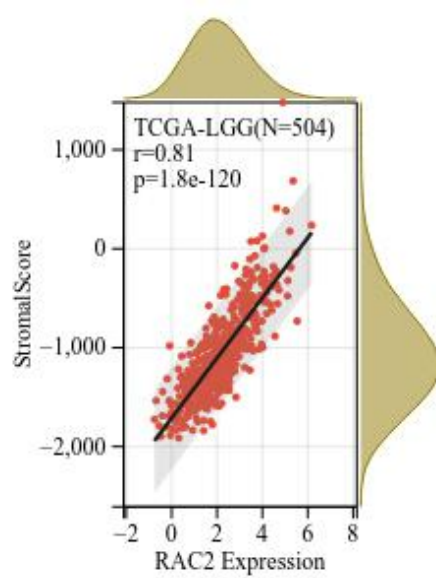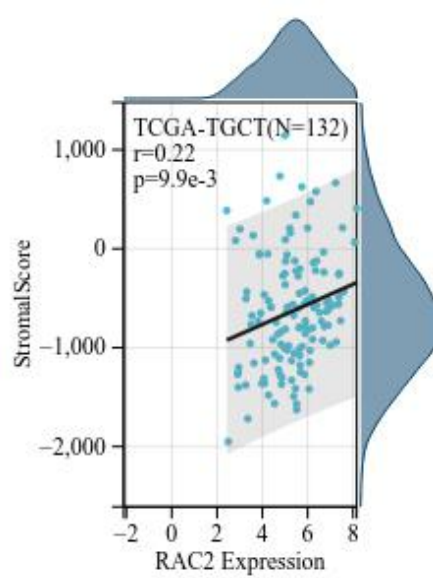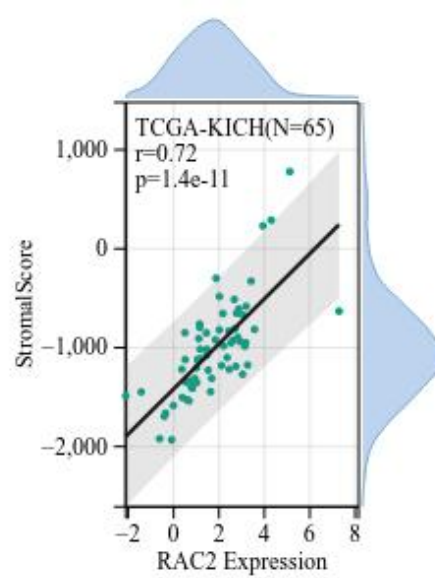

Supplement: Supplementary 2 — Figure S1: the 33 tumors in the TGCT database and their corresponding abbreviations. Figure S2: top three tumors with significant correlation between RAC2 and immune score: TGCT, LGG, and KICH. Figure S3: correlation of RAC2 expression and chemokines and receptors. Red indicates a positive correlation, while blue indicates a negative correlation. The dot plot showed the top 4 strongest associations. Figure S4: correlation of RAC2 expression and MHC molecules. Red indicates a positive correlation, while blue indicates a negative correlation. The dot plot showed the top 4 strongest associations. Figure S5-S10: expression of CD4+ T cells, CD8+ T cells, neutrophils, macrophages, dendritic cells, and natural killer cells in relation to individual tumors in each algorithm. [file 8485726.f2.zip › Figure S4 (1).pdf]

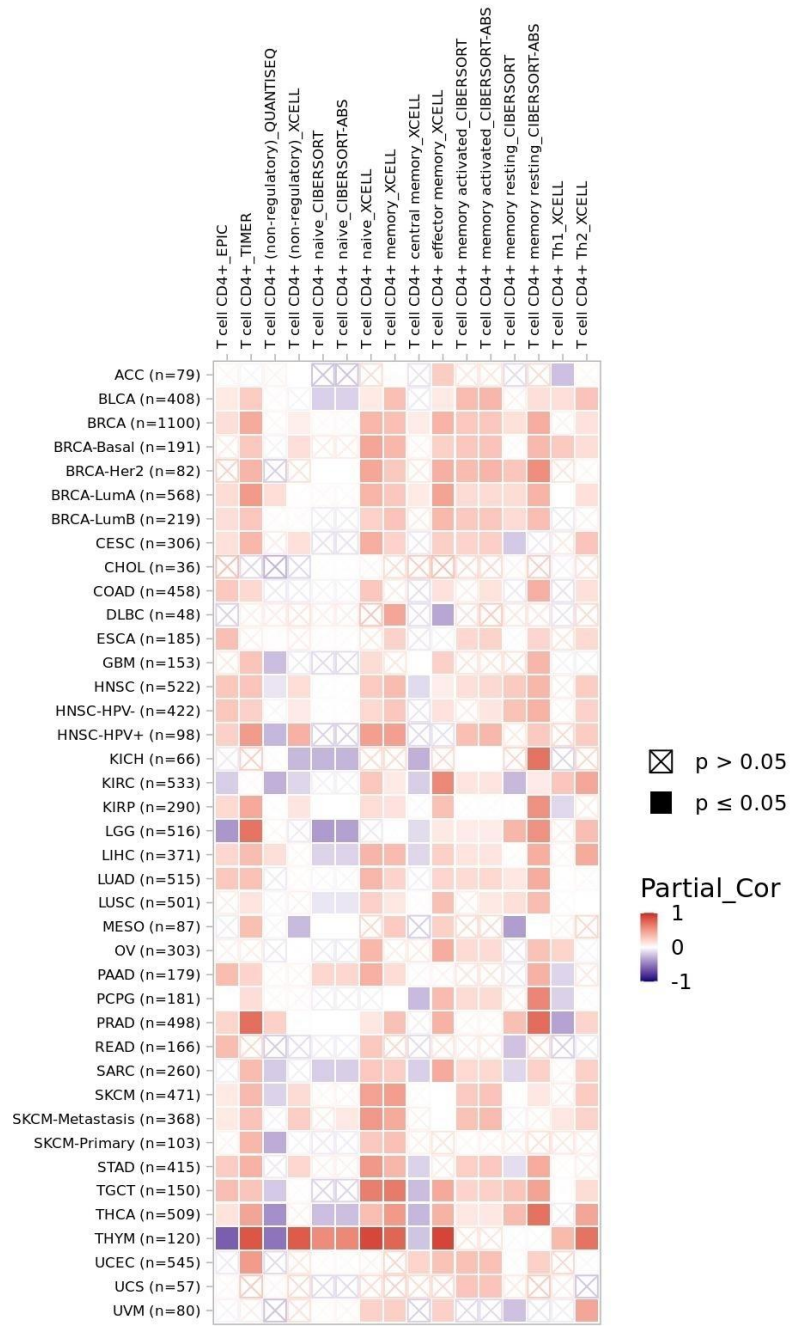

Supplement: Supplementary 2 — Figure S1: the 33 tumors in the TGCT database and their corresponding abbreviations. Figure S2: top three tumors with significant correlation between RAC2 and immune score: TGCT, LGG, and KICH. Figure S3: correlation of RAC2 expression and chemokines and receptors. Red indicates a positive correlation, while blue indicates a negative correlation. The dot plot showed the top 4 strongest associations. Figure S4: correlation of RAC2 expression and MHC molecules. Red indicates a positive correlation, while blue indicates a negative correlation. The dot plot showed the top 4 strongest associations. Figure S5-S10: expression of CD4+ T cells, CD8+ T cells, neutrophils, macrophages, dendritic cells, and natural killer cells in relation to individual tumors in each algorithm. [file 8485726.f2.zip › Figure S5 (1).pdf]

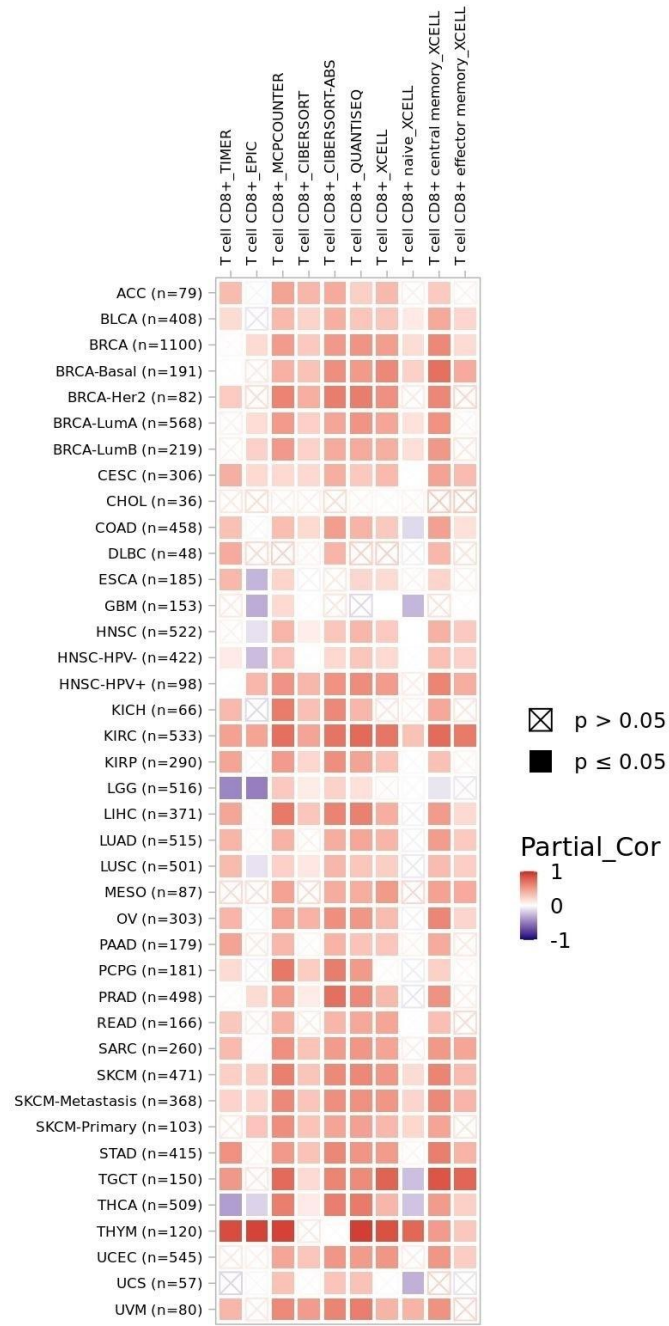

Supplement: Supplementary 2 — Figure S1: the 33 tumors in the TGCT database and their corresponding abbreviations. Figure S2: top three tumors with significant correlation between RAC2 and immune score: TGCT, LGG, and KICH. Figure S3: correlation of RAC2 expression and chemokines and receptors. Red indicates a positive correlation, while blue indicates a negative correlation. The dot plot showed the top 4 strongest associations. Figure S4: correlation of RAC2 expression and MHC molecules. Red indicates a positive correlation, while blue indicates a negative correlation. The dot plot showed the top 4 strongest associations. Figure S5-S10: expression of CD4+ T cells, CD8+ T cells, neutrophils, macrophages, dendritic cells, and natural killer cells in relation to individual tumors in each algorithm. [file 8485726.f2.zip › Figure S6 (1).pdf]

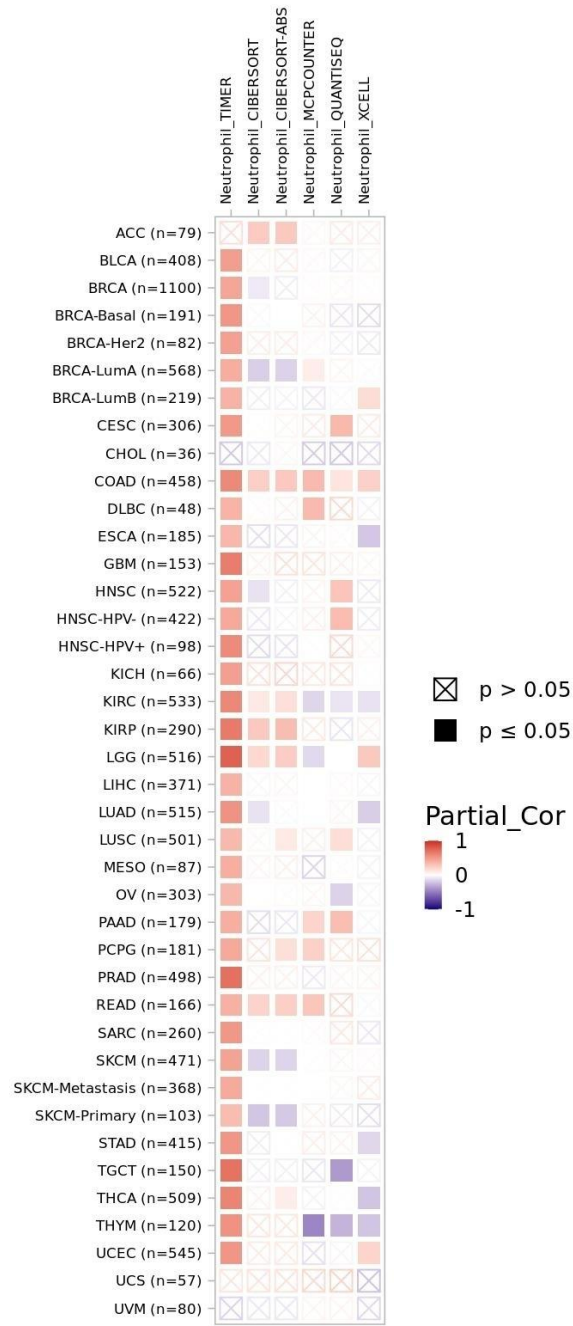

Supplement: Supplementary 2 — Figure S1: the 33 tumors in the TGCT database and their corresponding abbreviations. Figure S2: top three tumors with significant correlation between RAC2 and immune score: TGCT, LGG, and KICH. Figure S3: correlation of RAC2 expression and chemokines and receptors. Red indicates a positive correlation, while blue indicates a negative correlation. The dot plot showed the top 4 strongest associations. Figure S4: correlation of RAC2 expression and MHC molecules. Red indicates a positive correlation, while blue indicates a negative correlation. The dot plot showed the top 4 strongest associations. Figure S5-S10: expression of CD4+ T cells, CD8+ T cells, neutrophils, macrophages, dendritic cells, and natural killer cells in relation to individual tumors in each algorithm. [file 8485726.f2.zip › Figure S7 (1).pdf]

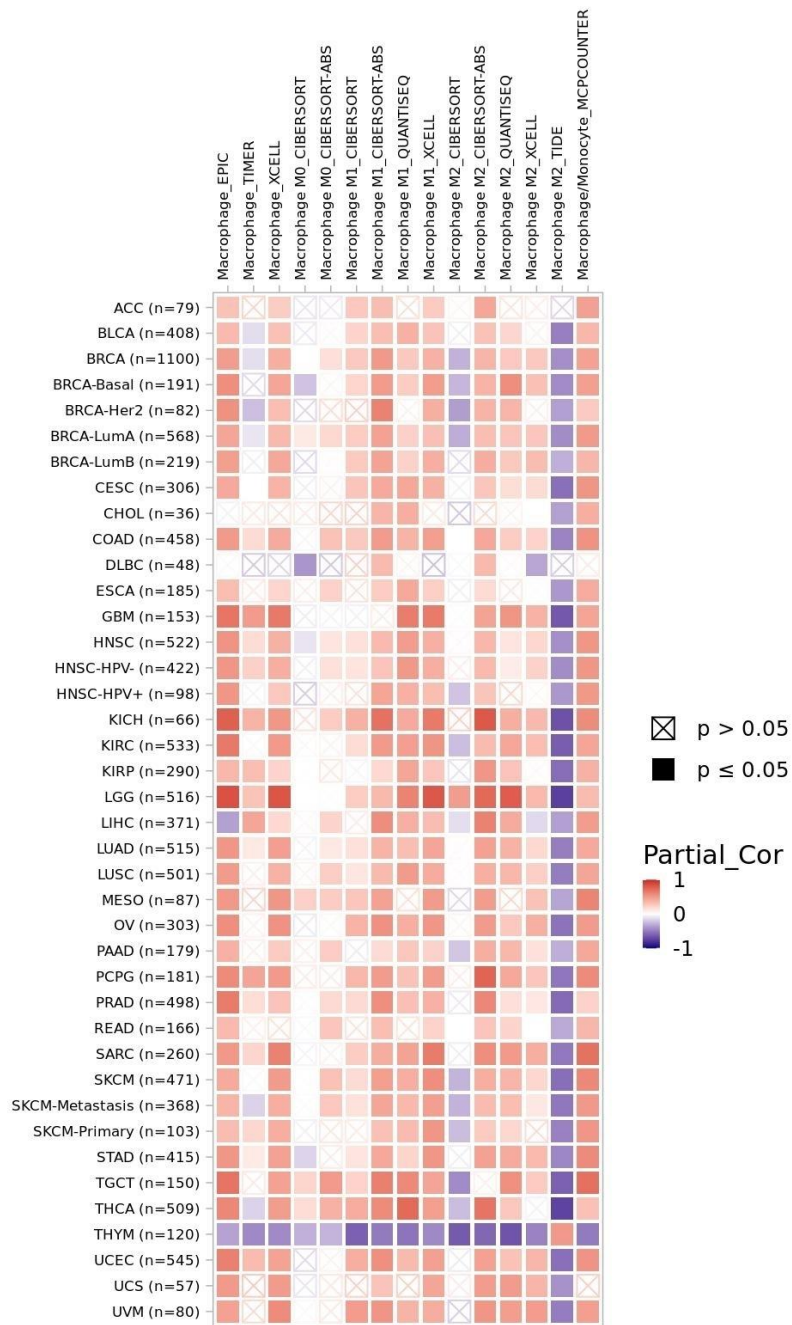

Supplement: Supplementary 2 — Figure S1: the 33 tumors in the TGCT database and their corresponding abbreviations. Figure S2: top three tumors with significant correlation between RAC2 and immune score: TGCT, LGG, and KICH. Figure S3: correlation of RAC2 expression and chemokines and receptors. Red indicates a positive correlation, while blue indicates a negative correlation. The dot plot showed the top 4 strongest associations. Figure S4: correlation of RAC2 expression and MHC molecules. Red indicates a positive correlation, while blue indicates a negative correlation. The dot plot showed the top 4 strongest associations. Figure S5-S10: expression of CD4+ T cells, CD8+ T cells, neutrophils, macrophages, dendritic cells, and natural killer cells in relation to individual tumors in each algorithm. [file 8485726.f2.zip › Figure S8.pdf]

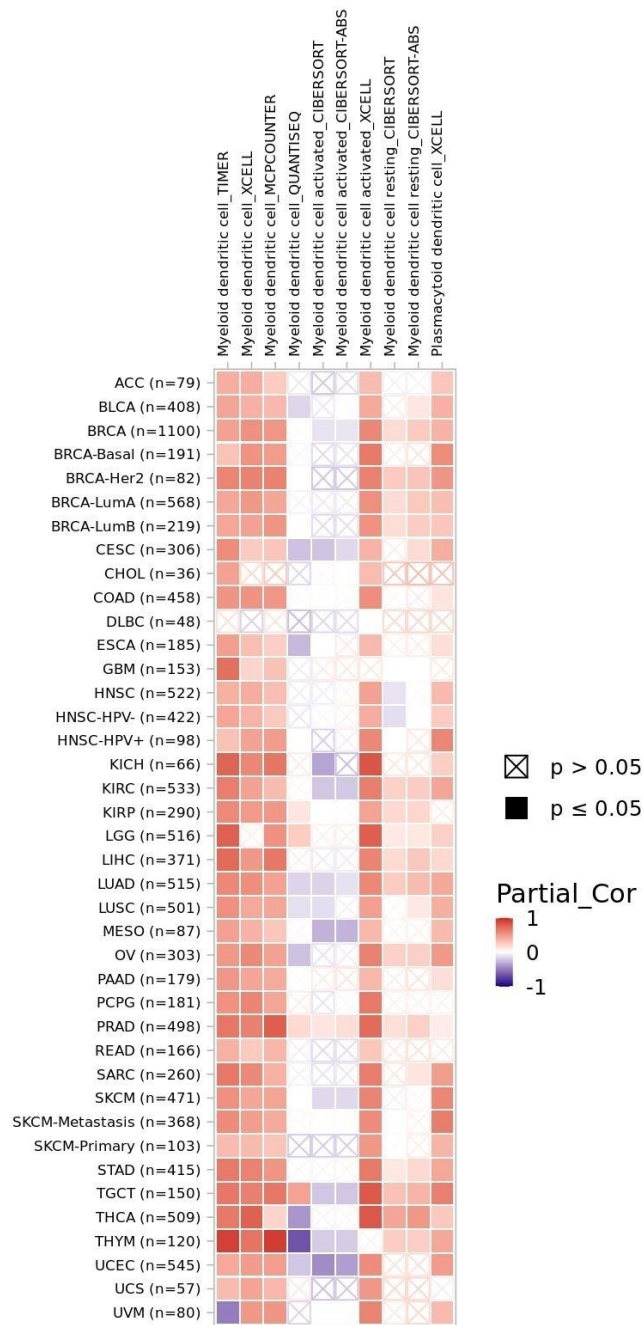

Supplement: Supplementary 2 — Figure S1: the 33 tumors in the TGCT database and their corresponding abbreviations. Figure S2: top three tumors with significant correlation between RAC2 and immune score: TGCT, LGG, and KICH. Figure S3: correlation of RAC2 expression and chemokines and receptors. Red indicates a positive correlation, while blue indicates a negative correlation. The dot plot showed the top 4 strongest associations. Figure S4: correlation of RAC2 expression and MHC molecules. Red indicates a positive correlation, while blue indicates a negative correlation. The dot plot showed the top 4 strongest associations. Figure S5-S10: expression of CD4+ T cells, CD8+ T cells, neutrophils, macrophages, dendritic cells, and natural killer cells in relation to individual tumors in each algorithm. [file 8485726.f2.zip › Figure S9.pdf]
