# Supplementary material for: Pancancer Analysis Revealed the Value of RAC2 in Immunotherapy and Cancer Stem Cell
Source: Stem Cells Int. 2023 May 12;2023:8485726. doi: 10.1155/2023/8485726 (PMC10198763; doi:10.1155/2023/8485726)
Supplement: Supplementary 2 — Figure S1: the 33 tumors in the TGCT database and their corresponding abbreviations. Figure S2: top three tumors with significant correlation between RAC2 and immune score: TGCT, LGG, and KICH. Figure S3: correlation of RAC2 expression and chemokines and receptors. Red indicates a positive correlation, while blue indicates a negative correlation. The dot plot showed the top 4 strongest associations. Figure S4: correlation of RAC2 expression and MHC molecules. Red indicates a positive correlation, while blue indicates a negative correlation. The dot plot showed the top 4 strongest associations. Figure S5-S10: expression of CD4+ T cells, CD8+ T cells, neutrophils, macrophages, dendritic cells, and natural killer cells in relation to individual tumors in each algorithm. [file 8485726.f2.zip › Figure S1 (1).pdf]

Abbr	Full name
TCGA-ACC	Adrenocortical carcinoma
TCGA-BLCA	Bladder Urothelial Carcinoma
TCGA-BRCA	Breast invasive carcinoma
TCGA-CESC	Cervical squamous cell carcinoma and endocervical adenocarcinoma
TCGA-CHOL	Cholangiocarcinoma
TCGA-COAD	Colon adenocarcinoma
TCGA-DLBC	Lymphoid Neoplasm Diffuse Large B-cell Lymphoma
TCGA-ESCA	Esophageal carcinoma
TCGA-GBM	Glioblastoma multiforme
TCGA-HNSC	Head and Neck squamous cell carcinoma
TCGA-KICH	Kidney Chromophobe
TCGA-KIRC	Kidney renal clear cell carcinoma
TCGA-KIRP	Kidney renal papillary cell carcinoma
TCGA-LAML	Acute Myeloid Leukemia
TCGA-LGG	Brain Lower Grade Glioma
TCGA-LIHC	Liver hepatocellular carcinoma
TCGA-LUAD	Lung adenocarcinoma
TCGA-LUSC	Lung squamous cell carcinoma
TCGA-MESO	Mesothelioma
TCGA-OV	Ovarian serous cystadenocarcinoma
TCGA-PAAD	Pancreatic adenocarcinoma
TCGA-PCPG	Pheochromocytoma and Paraganglioma
TCGA-PRAD	Prostate adenocarcinoma
TCGA-READ	Rectum adenocarcinoma
TCGA-SARC	Sarcoma
TCGA-STAD	Stomach adenocarcinoma
TCGA-SKCM	Skin Cutaneous Melanoma
TCGA-TGCT	Testicular Germ Cell Tumors
TCGA-THCA	Thyroid carcinoma
TCGA-THYM	Thymoma
TCGA-UCEC	Uterine Corpus Endometrial Carcinoma
TCGA-UCS	Uterine Carcinosarcoma
TCGA-UVM	Uveal Melanoma
